# Transcriptomic analysis reveals *Aspergillus oryzae* responds to temperature stress by regulating sugar metabolism and lipid metabolism

**DOI:** 10.1371/journal.pone.0274394

**Published:** 2022-09-12

**Authors:** Chunmiao Jiang, Jinxin Ge, Bin He, Zhe Zhang, Zhihong Hu, Yongkai Li, Bin Zeng

**Affiliations:** 1 Jiangxi Key Laboratory of Bioprocess Engineering and Co-Innovation Center for In-Vitro Diagnostic Reagents and Devices of Jiangxi Province, College of Life Sciences, Jiangxi Science and Technology Normal University, Nanchang, China; 2 College of Pharmacy, Shenzhen Technology University, Shenzhen, China; Central University of Punjab, INDIA

## Abstract

*Aspergillus oryzae* is widely used in industrial applications, which always encounter changes within multiple environmental conditions during fermentation, such as temperature stress. However, the molecular mechanisms by which *A*. *oryzae* protects against temperature stress have not been elucidated. Therefore, this study aimed to characterize the fermentative behavior, transcriptomic profiles, and metabolic changes of *A*. *oryzae* in response to temperature stress. Both low and high temperatures inhibited mycelial growth and conidial formation of *A*. *oryzae*. Transcriptomic analysis revealed that most differentially expressed genes (DEGs) were involved in sugar metabolism and lipid metabolism under temperature stress. Specifically, the DEGs in trehalose synthesis and starch metabolism were upregulated under low-temperature stress, while high temperatures inhibited the expression of genes involved in fructose, galactose, and glucose metabolism. Quantitative analysis of intracellular sugar further revealed that low temperature increased trehalose accumulation, while high temperature increased the contents of intracellular trehalose, galactose, and glucose, consistent with transcriptome analysis. In addition, most DEGs involved in lipid metabolism were significantly downregulated under low-temperature stress. Furthermore, the metabolomic analysis revealed that linoleic acid, triacylglycerol, phosphatidylethanolamine, and phosphoribosyl were significantly decreased in response to low-temperature stress. These results increase our understanding of the coping mechanisms of *A*. *oryzae* in response to temperature stress, which lays the foundation for future improvements through genetic modification to enhance *A*. *oryzae* against extreme temperature stress.

## Introduction

*Aspergillus oryzae* is an important filamentous fungus widely used in traditional fermented food products, such as sake, miso, and soybean sauce in East Asian [1, 2]. During fermentation, *A*. *oryzae* is exposed to multiple environmental stresses, such as high salinity, ethanol, and varying temperatures. Temperature is the most important environmental factor that affects the growth and activity of *A*. *oryzae* and directly affects the activity of enzymes digesting the substrates, thus, controlling the production of compounds and the rate of fermentation [2, 3]. *A*. *oryzae* cells encounter rapid and large temperature changes, hence are being optimized to grow at certain temperatures dependent on the production process. The optimal temperature for *A*. *oryzae* growth is 30~35°C [4], with temperatures above or below this range adversely affecting its growth. However, to effectively improve the relative content of alcohols, esters, phenols, and other substances that affect the flavor of fermentation, the temperature varies from 15 to 45°C during the whole industrial fermentation. Although *A*. *oryzae* can still grow and/or ferment well outside its optimal growth temperature and displays strong adaptability to different temperatures, its underlying molecular mechanisms under temperature stress have not been reported. Therefore, it is of substantial significance to understand these mechanisms, which can be exploited to engineer *A*. *oryzae* well adapted and tolerant to extreme temperatures to enhance the production efficiency in its industrial applications.

The ability of microorganisms to adapt to different environmental temperatures has attracted considerable attention, although the mechanism that underlies this phenomenon is not well understood. For growth and/or fermentation to occur at low or high temperatures, all cell constituents must be stable and functional under these conditions. Therefore, the characterization of the changes in cells after they have been subjected to various stimuli is crucial to comprehending the adaptation of cells to change conditions. For example, the adjustment of thermophiles to higher temperatures encompasses a range of molecular mechanisms that do not appear to be owing to any single factor. One option is a change in the composition of phospholipids, including phosphatidylcholine (PC), phosphatidylethanolamine (PE), phosphatidylinositol (PI), phosphatidylserine (PS), phosphatidylglycerol (PG), and phosphatidic acid (PA), or the unique lipids of inositol phosphoceramide and mannosylinositol phosphoceramide to promote the swift adjustment to temperature changes [5]. The lipids serve as temperature sensors, which initiate crucial cellular responses and developmental programs in microorganisms in response to temperature changes [6]. In addition, many microorganisms synthesize important osmotically active compounds, such as sorbitol and trehalose, which stabilize proteins, cell walls, and membranes against several environmental stresses, particularly extreme temperatures [7–9]. For example, trehalose and PA are increased in thermophilic fungi under heat shock, while under cold stress, there is no glycerol accumulation in the mycelia followed by a decrease in trehalose concentration [10]. In addition, the sugar, glycolysis, and amino acid metabolisms are significantly altered in response to high-temperature stress in *Lentinula edodes* [11]. However, the protective biochemical mechanisms of *A*. *oryzae* in response to temperature stress are poorly understood. Understanding these mechanisms is critical for the survival and adaptation of *A*. *oryzae* to different temperatures during the fermentation of foods.

Fungal cells reprogram the expression of genes that are essential for the adaptation to environmental stresses. Low temperature or heat stress destroys the stability and fluidity of membranes, which determine the sensitivity/tolerance of cells toward stress conditions, directly affecting many membrane-linked physiological processes [12]. To overcome the hurdles imposed by temperature stress, fungi change the expression of genes involved in cell membrane function, the production of cold or heat shock proteins and other molecular strategies to maintain homeostasis [13]. In *Candida albicans* and *Saccharomyces cerevisiae*, approximately 10~20% of the genes are induced in response to heat shock stress [14, 15]. The heat shock protein 90 is exquisitely sensitive to elevated temperature as a wide range of client proteins in protein folding that is involved in signaling and heat stress responses [16]. In addition, abnormally high/low temperatures trigger a transient expression of stress-protective genes involved in lipid and carbohydrate metabolism, which function in generation of energy to defend against temperature stress. For example, the genes involved in energy and trehalose metabolism and those encoding molecular chaperones are significantly induced, while the transporters and genes associated with purine metabolism are downregulated in response to a temperature change from 28 to 41°C in *S*. *cerevisiae* [17]. However, the specific genes involved in these processes and their expression pattern under different temperature conditions in *A*. *oryzae* remain unclear.

To better understand the effect of low and high temperatures on the *A*. *oryzae* growth and activity, its responses and changes in gene expression under different temperatures (22, 30 and 42°C) at the whole-genome level were analyzed through RNA-sequencing (RNA-seq). The genome sequencing of *A*. *oryzae* strain 3.042 facilitated a global view of the expression of genes in response to various stimuli [18]. In this study, the comparisons of gene expression between the samples outlined the variation in the differentially expressed genes (DEGs) under low- or high-temperature stress, illustrating the activated metabolic pathways in response to temperature stress. The findings in this study provide a theoretical basis for understanding the underlying molecular mechanism in *A*. *oryzae* in response to low- or high- temperature stress, which lays the foundation for molecular breeding of new *A*. *oryzae* strains that are resistant to low or high temperatures.

## Materials and methods

### Effects of temperature on *A*. *oryzae*

*Aspergillus oryzae* strain 3.042 (CICC 40092) was used in this study. The *A*. *oryzae* inoculum was prepared by inoculating conidia in a freshly potato dextrose agar (PDA) medium, then incubated for 72 h at 30°C to harvest the conidia. The concentration of *A*. *oryzae* conidia was determined using a haemocytometer. A volume of 2 μL freshly prepared conidia suspension containing approximately 1 × 10^7^ conidia was dotted on the PDA medium and incubated at 22, 25, 30, 35, and 42°C for 72 h. After 72 h, the colony sizes were determined by measuring the colony diameter. Next, the spores were collected from the colonies and suspended in sterile distilled water to obtain the spore suspensions. The number of spores in each suspension was then counted using a haemocytometer. In addition, 100 μL of freshly diluted 1 × 10^7^ conidia suspension were spread on PDA medium covered with cellophane, and incubated at 22, 25, 30, 35, and 42°C for 72 h. After 72 h, the fungal mycelia were collected, dried overnight, and then the biomass was determined. Each treatment was replicated thrice.

### Preparation of transcriptome samples and RNA sequencing

A total of 100 μL of freshly diluted 1 × 10^7^ *A*. *oryzae* conidia were inoculated PDA medium covered with cellophane, and incubated at 22, 30, and 42°C for 72 h. The fungal mycelia were collected for total RNA extraction using an Omega fungal RNA kit (Omega Bio-Tek, Georgia, USA). The purity and concentration of total extracted RNA were determined by NanoDrop 2000 spectrophotometer and Qubit 2.0 fluorometer (Thermo Scientific, Wilmington, DE, USA). In addition, the integrity of the total extracted RNA was analyzed by Agilent 2100 bioanalyzer (Agilent Technologies, Palo Alto, CA, USA). Next, RNA samples per treatment were pooled to create equal quantities of RNA samples per treatment for cDNA library construction to ensure reliability and reproducibility. The mRNA was enriched from the total RNA using oligo (dT) magnetic beads, then digested into short pieces using the fragmentation buffer at 94°C for 5 min. Using the mRNA fragments as templates, the first strand of cDNA was synthesized through random hexamer primers, followed by the second-strand cDNA synthesis using DNA polymerase I and RNase H. After end-repair and adaptor ligation, the products were amplified by PCR and further purified by QIAquick PCR purification kit following the manufacturer’s protocol to create a cDNA library. Then the libraries were sequenced using the Illumina HiSeq 2500 platform (Illumina, San Diego, CA, USA) at Novogene Bioinformatics Technology Co., Ltd (Beijing, China). A total of 125 bp paired-end reads were obtained. The sequencing data were deposited in the NCBI/SRA database (Bioproject: PRJNA774152; SRA: SRR16556442, SRR16556443, and SRR16556444).

### Mapping reads to the reference genome and normalized gene expressions

After sequencing, the raw reads were cleaned by removing low-quality reads, adaptor sequences and reads with N (uncertain bases) exceeding 10%. The obtained clean reads were aligned to the *A*. *oryzae* 3.042 genome (https://www.ncbi.nlm.nih.gov/genome/526?genome_assembly_id=29881) using Tophat software (v2.0.7; http://ccb.jhu.edu/software/tophat/index.shtml). In addition, the Q20, Q30, GC-content and sequence duplication rates of the clean reads were calculated. To screen for the DEGs between treatments, the transcript abundances were normalized using the fragments per kilobase of transcript per million mapped fragments (FPKM) metric. The differential gene expression analysis was performed by DESeq2 software [19] between two groups. The genes with a false discovery rate (FDR) below 0.05 and absolute fold change ≥1 were considered DEGs [20]. All DEGs were mapped to GO terms in the Gene Ontology (GO) database (http://www.geneontology.org/) to calculate gene numbers for every term and the significantly enriched GO terms in the DEGs compared to the genome background were defined using a hypergeometric test. The adjusted p-value of FDR ≤0.05 was set as a threshold. The GO terms meeting this condition were defined as significantly enriched in DEGs. Pathway enrichment analyses were based on the Kyoto Encyclopedia of Genes and Genomes (KEGG) database [21, 22]. The significantly enriched metabolic pathways or signal transduction pathways in DEGs were identified by comparing then to the whole genome background. The adjusted p-value of FDR ≤0.05 was set as the threshold. Pathways meeting this condition were defined as significantly enriched pathways in DEGs.

### Alternative splicing analysis

The frequency and types of alternative splicing events were identified using the rMATS software (http://rnaseq-mats.sourceforge.net/index.html). The results of TopHat included all alternative splicing information and junction structure were determined as follows: first, alternative splicing sites with less than five reads were filtered out. The remaining alternative splicing sites were mapped to known alternative splicing sites (1 bp error was allowed) to identify known alternative splicing sites. Finally, the unmapped new alternative splicing sites were classified into five categories in our study, SE (skipped exon), RI (retained intron), MXE (mutually exclusive exon), A5SS (alternative 5’ splice site), and A3SS (alternative 3’ splice site).

### qRT-PCR for analyzing the expression profiles of DEGs involved in sugar or lipid metabolism pathways

To confirm the reliability of the gene expression data, quantitative reverse transcription-PCR (qRT-PCR) was conducted for 20 genes involved in sugar or lipid metabolism under different temperature treatments. The primers used for qRT-PCR are listed in S1 Table. The total RNA was extracted using the Omega fungal RNA kit (Omega Bio-Tek, Georgia, USA) according to the manufacturer’s instructions. A total 1 μg of the extracted RNA was reverse-transcribed into cDNA using PrimeScript™ RT reagent with the gDNA Eraser kit (TaKaRa, Dalian, China) following the manufacturer’s instructions. The gene expression levels were determined by qRT-PCR on a Bio-rad CFX96 Touch instrument (Bio-Rad, USA) using TB Premix Ex Taq II (TaKaRa) according to the manufacturer’s instructions. The histone *H1* gene was used as the reference gene in qRT-PCR analysis. Data were analyzed using Bio-rad CFX96 software and the 2^-ΔΔCT^ method [23].

### Quantification of intracellular sugar contents by HPLC

*A*. *oryzae* mycelia incubated at different temperatures were collected as described under the preparation of transcriptome samples and RNA sequencing above. The sugar components in the mycelia were extracted according to the method described by Li et al. [24]. The presence and contents of sugar (galactose, fructose, glucose, sorbitol, and trehalose) were analyzed by high-performance liquid chromatography (HPLC) (Thermo Scientific™ UltiMate TM 3000) according to the method of Li et al. [24]. The samples were isolated on the Innoval NH_2_ column (4.6 mm × 300 mm, 5 μm, Thermo Scientific™) at room temperature. HPLC was conducted using a mobile phase (acetonitrile:water = 80:20 (V/V)), 20 μL sample volume, 0.5 mL/min flow rate, 80°C column temperature, and 40°C detection temperature. Next, the mixed standard solution of galactose, fructose, glucose, sorbitol, and trehalose (2 mg/mL) was prepared, and the sample injection was repeated six times. The six identical samples were then weighed, and the content of sugar components in each sample was calculated according to the peak area. Finally, the standard deviation of the standard solution was calculated according to the peak area of each standard sample.

### The effects of exogenous sugar on the growth of *A*. *oryzae* under different temperature stresses

The effects of exogenous sugar on *A*. *oryzae* under low and high temperature stress were determined using Czapek-Dox (CD) medium (0.2% NaNO_3_, 0.1% KH_2_PO_4_, 0.05% MgSO_4_, 0.05% KCl, 0.05% NaCl, 0.002% FeSO_4_, and 1.8% agar at pH 5.5) with a final concentration (4 and 8 g/100 mL) of starch, fructose, or galactose. The CD medium without sugar was used as the control. A 2 μL volume of freshly prepared *A*. *oryzae* suspension containing 1 × 10^7^ conidia was inoculated on the medium and incubated for 96 h at 22, 30, and 42°C. The colony diameters and the number of spores were measured as described under effects of temperature treatment on *A*. *oryzae* above. Each treatment was performed in triplicate.

### Determination of the relative lipid contents

*A*. *oryzae* mycelia cultured at different temperatures were collected as described under the preparation of transcriptome samples and RNA sequencing above. Total lipids were extracted from 100 mg of freeze-dried mycelial samples and analyzed using the liquid chromatograph mass spectrometer (LC-MS) [25]. The raw data were obtained by LipidSearch software v4.0 (Thermo Scientific) [26]. Each lipid was annotated to obtain the mass to charge ratio (m/z) data matrix, retention time (rt) and peak intensity. Next, the lipid peaks were identified, filtered, and aligned using the LipidSearch software. The main parameters were set as follows: R.T.Tolerance = 0.25, and m-Score Threshold = 2. All the peak intensities were batch normalized to the total spectral intensity to compare the data of different orders of magnitude.

### Statistical analyses

All experiments were performed in triplicate. All data were presented as the mean ± standard deviation (SD). Data were analyzed by a nested one-way analysis of variance (ANOVA), and the means from the same treatment were separated using the least significant difference test (LSD). Differences between the sample means were analyzed by Duncan’s multiple range test at *P*<0.05 using SPSS for Windows, Standard Version 8.0.

## Results

### Effects of different temperatures on the growth of *A*. *oryzae*

**Fig 1:**
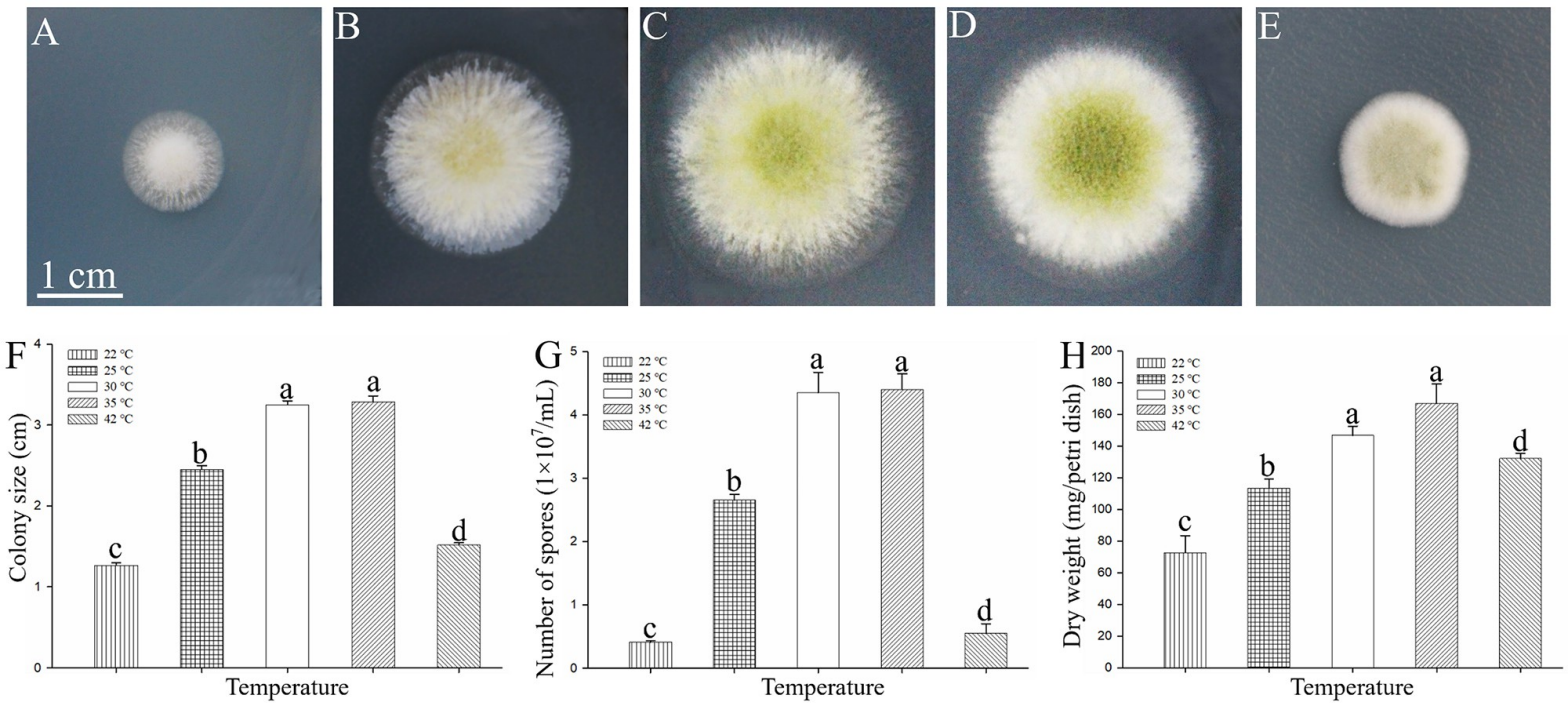
The hyphal growth and differentiation of *A*. *oryzae* under different temperatures for 72 h. (A-E) The phenotypes of *A*. *oryzae* on PDA media were incubated at different temperature, including 22, 25, 30, 35, and 42°C for 72 h from left to right. (F) The colony sizes based on the diameter of the colonies. (G and H) The number of spores and dry biomass of *A*. *oryzae* mycelia under different temperature treatments. All values are mean ± SD of three replicates per treatment. The different lower-case letters indicate significant differences (*P*<0.05, n = 3), while the same letters indicate insignificant differences.

A preliminary study involving different temperature treatments was conducted to analyze the effects of subjecting *A*. *oryzae* to temperatures outside the optimal for its growth and development. Exposing *A*. *oryzae* to temperatures above or below the optimal range (30~35°C), specifically 22 and 42°C, significantly inhibited mycelial growth (Fig 1A–1E). In addition, the colony size, spore numbers, and the dry biomass of *A*. *oryzae* mycelia were significantly reduced at 22, 25, and 42°C compared to the control (CK, 30°C) (Fig 1F–1H). Moreover, the exposure to low and high temperatures remarkably inhibited the formation and development of spores (Fig 1A–1E).

### Transcriptome overview of *A*. *oryzae* under temperature stress

To characterize the changes in global gene expression during *A*. *oryzae* growth under different temperatures, three libraries were constructed using *A*. *oryzae* incubated at 22, 30, and 42°C. Each treatment had three biological replicates (LT_1, 2, 3: incubated at 22°C; CK_1, 2, 3: incubated at 30°C, and HT_1, 2, 3: incubated at 42°C). A total of 49.62, 49.61, and 45.03 million clean reads per library were obtained at 22, 30, and 42°Cafter removing low quality reads and adaptor sequences, respectively (Table 1). The clean reads aligned to the whole reference genome sequence mapped ≥ 93% of the clean reads for each library to the genome, which implied that the transcriptome data were suitable for further analysis. A total of 11,563, 12,070, and 12,041 genes were identified in the high-quality clean reads at 22, 30, and 42°C, among which 972, 1,045, and 1,031 were new genes, respectively (Table 1). These new genes will contribute to the re-annotation of the *A*. *oryzae* genome. Finally, a total of 12,974 unigenes, including 1,348 (10.39%) new genes and 11,626 (89.61%) known genes, were obtained.

**Table 1:** Summary of the sequencing data and gene numbers of *A*. *oryzae* transcriptome under three temperature treatments.

Samples	LT	CK	HT
Raw reads	51,164,648	51,134,962	46,240,702
Clean reads	49,623,648	49,611,004	45,033,506
Uniquely mapped	46,763,355 (94.24%)	46,356,962 (93.44%)	43,024,561 (95.54%)
Multiple mapped	135,527 (0.27%)	143,244 (0.29%)	134,601 (0.3%)
Total mapped	46,898,882 (94.51%)	46,500,206 (93.73%)	43,159,162 (95.84%)
Known gene number	10,591(91.59%)	11,025(91.34%)	11,010 (91.44%)
New gene number	972(8.41%)	1,045(8.66%)	1,031 (8.56%)
All gene number	11,563	12,070	12,041

Note: LT, samples incubated at 22°C. CK, samples incubated at 30°C. HT, samples incubated at 42°C.

### Alternative splicing analysis

**Fig 2:**
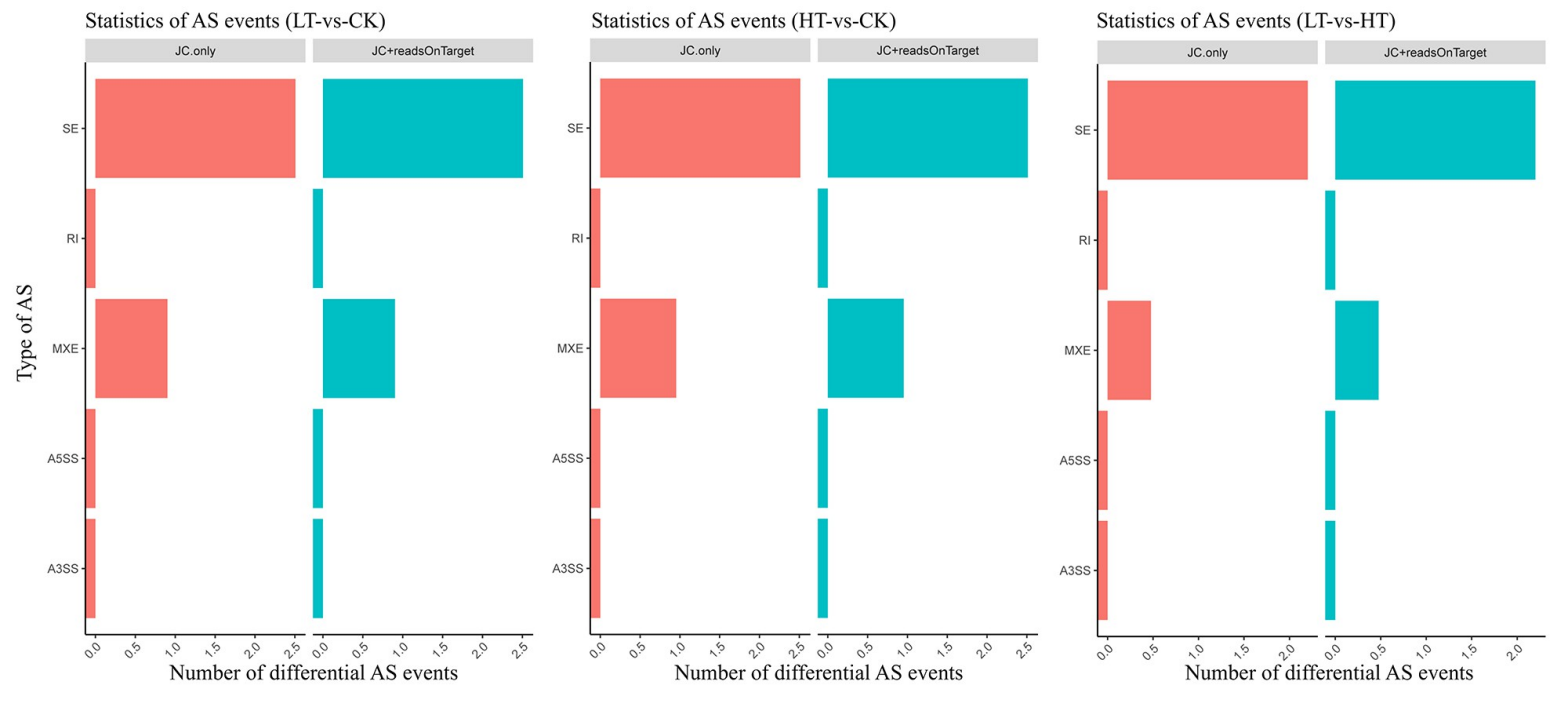
Different types of alternative splicing (AS) events among the comparison groups subjected to different temperatures. The horizontal axis indicates the type of alternative splicing, while the vertical axis indicates the number of differential alternative splicing events. AS, alternative splicing. JC, junction count. SE, skipped exon. RI, retained intron. MXE, mutually exclusive exon. A5SS, alternative 5’ splice site (junctions start inside an exon and end up with the initiation site of another exon). A3SS, alternative 3’ splice site (junctions start from an exon termination site and end up inside another exon). LT, samples incubated at 22°C. CK, samples incubated at 30°C. HT, samples incubated at 42°C.

Alternative splicing analysis is pivotal in identifying and quantifying differentially spliced transcripts for transcriptome analysis [27]. In this study, each comparison group was considered a unit in the analysis of the alternative splicing events, and the types and numbers of alternative splicing were counted. The different types of alternative splicing events were differentially analyzed. Junction count (JC) and reads on the target and JC were used to calculate the expression of each type of alternative splicing event. Finally, the alternative splicing sites identified in the LT-vs-CK, HT-vs-CK, and LT-vs-HT comparison groups were classified into five categories. The alternative splicing sites analysis revealed that the three comparison groups were highly similar, which confirmed the relative consistency among the replicates (Fig 2). In addition, the alternative splicing events at different temperatures showed a similar trend, and junctions that started at and ended up in the area between the genes were highly represented, followed by junctions that started at the 5’ end of the exon initiation sites and ended with another exon. These results suggested that the types and numbers of alternative splicing events in *A*. *oryzae* were not altered at the optimal, low, and high-temperature environments.

### DEGs in response to various temperature stresses

**Fig 3:**
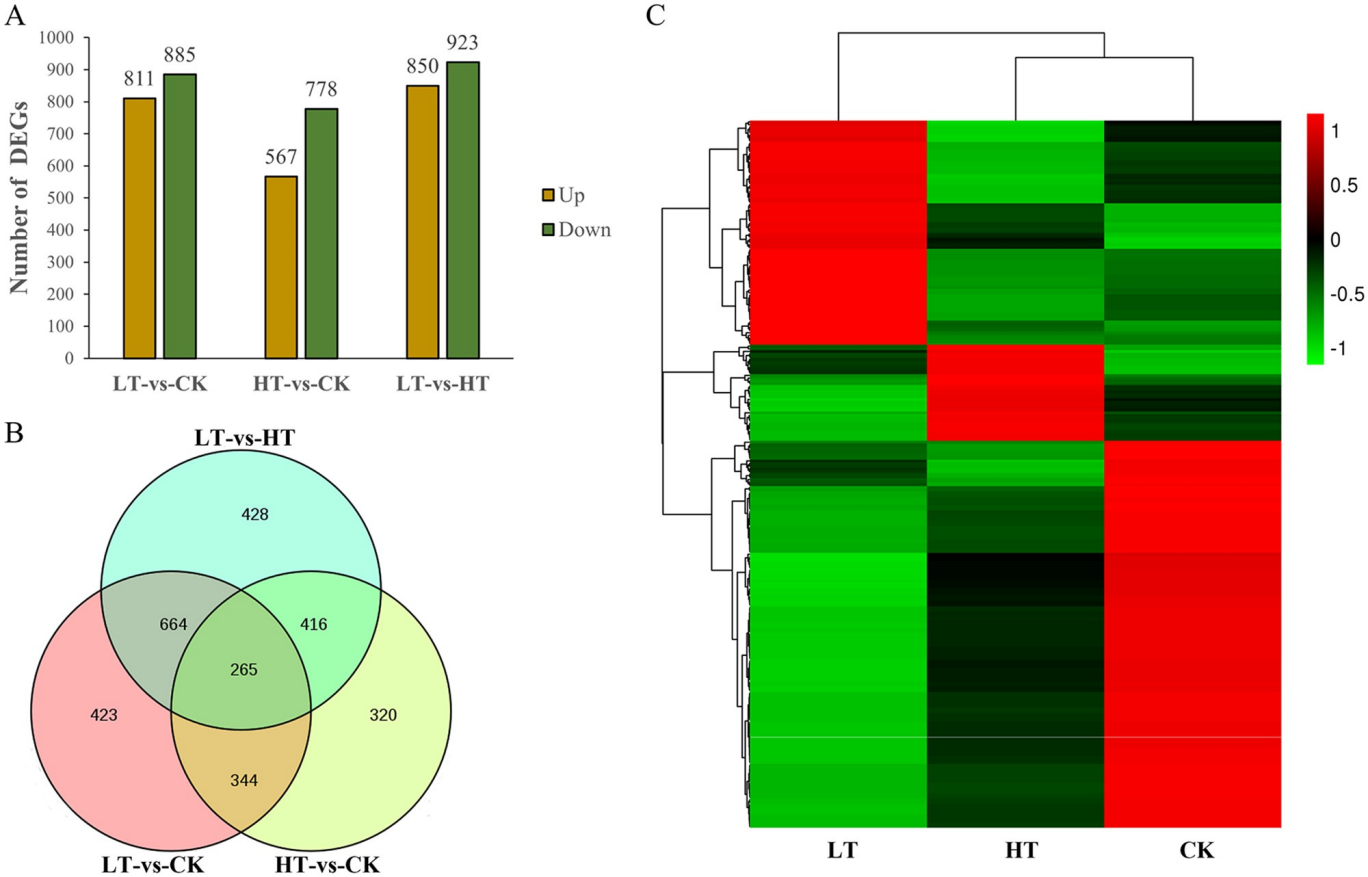
Analysis of the DEGs in *A*. *oryzae* incubated at different temperatures. (A) The distribution of DEGs between each pair of compared transcriptomes. (B) Venn diagram of DEGs *A*. *oryzae* incubated at different temperatures. The Venn diagram was generated using Venny 2.1 software (https://bioinfogp.cnb.csic.es/tools/venny/index.html). (C) A heatmap of 265 co-expressed DEGs in three temperature treatments. Red and green represent the upregulated and downregulated genes, respectively. The heatmap was generated using HemI_windows_1_0 software (http://hemi.biocuckoo.org/down.php). LT, samples incubated at 22°C. CK, samples incubated at 30°C. HT, samples incubated at 42°C.

A total of 1,696 genes were differentially expressed between the LT and CK groups, among which 811 (48%) were upregulated and 885 (52%) were downregulated (Fig 3A). There were 1,345 DEGs between the HT and CK groups, among which 567 (42%) were upregulated and 778 (58%) were downregulated. In addition, 1,773 DEGs were identified between the LT and HT groups, including 850 upregulated and 923 downregulated genes. The distribution of the DEGs is shown using a Venn diagram in Fig 3B. A total of 265 DEGs, including 170 downregulated and 95 upregulated genes in LT-vs-CK and 207 downregulated and 58 upregulated in HT-vs-CK, were commonly shared among the three compared groups. In addition, 423 specific DEGs in LT-vs-CK and 320 specific DEGs in HT-vs-CK might be involved in the adaptation to low and high temperatures, respectively. Interestingly, the expression level of each gene among the 265 DEGs displayed a contrasting trend between the low and high-temperature treatments (Fig 3C), indicating that *A*. *oryzae* employs different molecular mechanisms to adapt to low and high temperatures. The detailed expression levels and annotation of the 265 DEGs are shown in the S2 Table.

**Fig 4:**
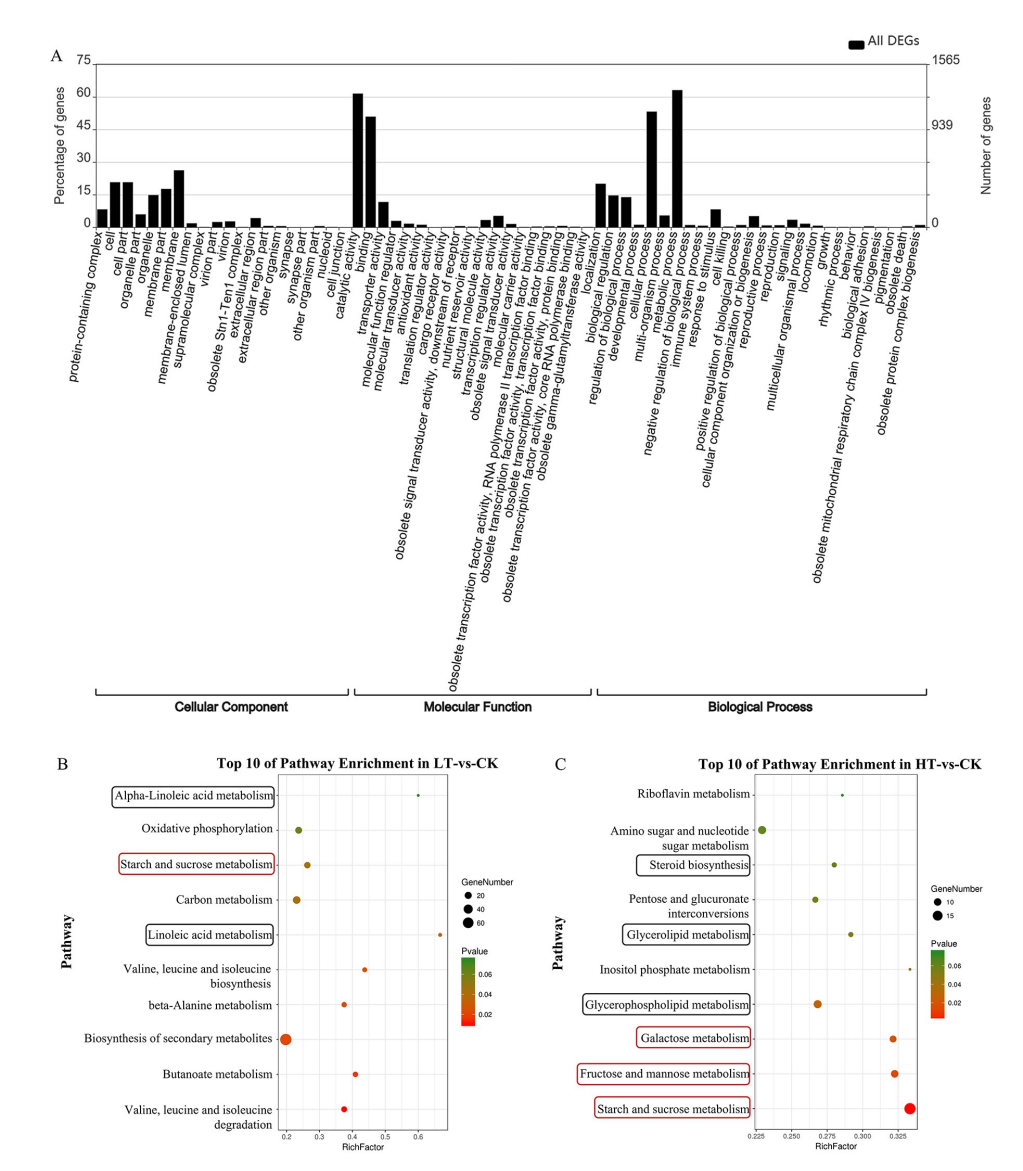
GO and KEGG pathway enrichment analyses of the DEGs under different temperature stresses. (A) The enriched GO terms of all the DEGs in response to temperature stress. (B, C) Top 10 enriched pathways in LT-vs-CK and HT-vs-CK, respectively. The black boxes represent the lipid metabolic pathways, while the red boxes represent the sugar metabolic pathways. GO, Gene Ontology. KEGG, Kyoto Encyclopedia of Genes and Genomes. DEGs, differentially expressed genes. LT, samples incubated at 22°C. CK, samples incubated at 30°C. HT, samples incubated at 42°C.

The GO functional enrichment analysis revealed that the significantly regulated genes were primarily related to the metabolic process (GO:0008152), catalytic activity (GO:0003824), cellular process (GO:0009987), binding (GO:0005488), and membrane (GO: 0016020) (Fig 4A). In addition, 774 DEGs were related to response to stimulus GO term (GO:0050896), directly involved in temperature stress (Fig 4A). Further, analysis of the response to stimulus GO term in LT-vs-CK and HT-vs-CK comparison groups revealed that there were 69 downregulated and 32 upregulated genes in response to low-temperature stress that were related to lipid pathway, environmental information processing and signaling pathway (S1 and S2 Figs, S3 Table). In addition, there were 84 DEGs, including 63 downregulated and 21 upregulated genes, in response to high-temperature stress (S4 Table). Most of these DEGs were related to lipid metabolism, environmental information processing or signaling processes. These results indicated that the incubation of *A*. *oryzae* at 22 or 42°C adversely affects its growth, and the DEGs could be involved in its adaptation to low or heat stress conditions. The top 10 enriched pathways in LT-vs-CK and HT-vs-CK are presented in Fig 4B and 4C. The DEGs were enriched in pathways involved in lipid metabolism (linoleic acid metabolism, steroid biosynthesis, glycerolipid metabolism, and glycerophospholipid metabolism) and sugar metabolism (starch and sucrose metabolism, galactose metabolism, and fructose and mannose metabolism) (Fig 4B and 4C), implying that these metabolic activities play an important role in response to low- or high-temperature stress. The qRT-PCR analysis of 20 genes involved in lipid and sugar metabolism was consistent with the transcriptome analysis (S3 Fig), which confirmed the reliability and availability of DEGs in the transcriptome analysis.

### DEGs involved in sugar metabolism in response to temperature stress

**Fig 5:**
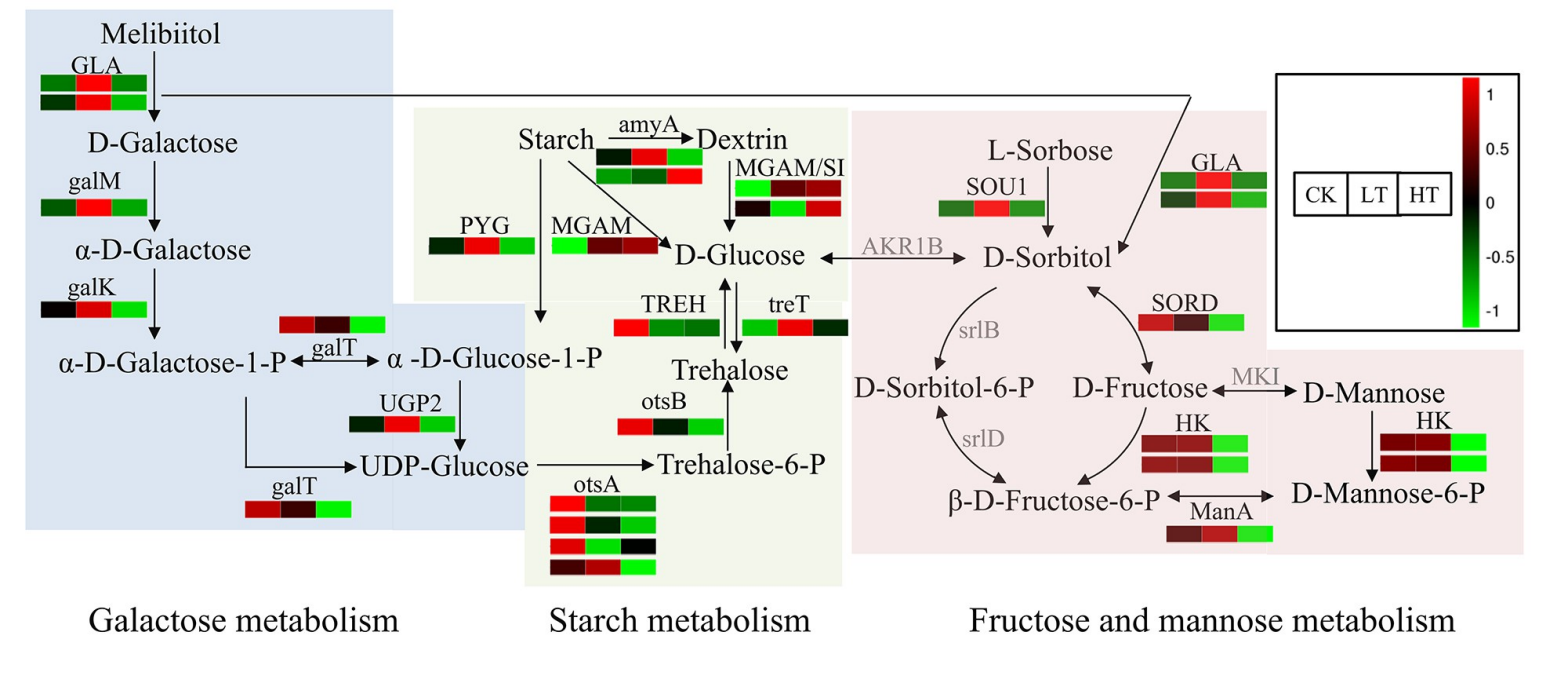
KEGG enrichment analysis of DEGs encoding key enzymes involved in the sugar metabolic pathways. Red and green indicate that the gene expressions were upregulated and downregulated, respectively. Grey indicates insignificant gene expressions. GLA, alpha-galactosidase. galM, aldose 1-epimerase. galK, galactokinase. galT, UDPglucose—hexose-1-phosphate uridylyltransferase. UGP2, UTP—glucose-1-phosphate uridylyltransferase. amyA, alpha-amylase. MGAM, maltase-glucoamylase. SI, sucrase-isomaltase. treT, trehalose synthase. otsA, trehalose 6-phosphate synthase. otsB, trehalose 6-phosphate phosphatase. TREH, alpha, alpha-trehalase. SOU1, sorbose reductase. SORD, L-iditol 2-dehydrogenase. HK, hexokinase. srlD, sorbitol-6-phosphate 2-dehydrogenase. srlB, glucitol/sorbitol PTS system EIIA component. MKI, mannose isomerase. ManA, mannose-6-phosphate isomerase. KEGG, Kyoto Encyclopedia of Genes and Genomes. DEGs, differentially expressed genes. LT, samples incubated at 22°C. CK, samples incubated at 30°C. HT, samples incubated at 42°C.

Sugar metabolism is related to several types of carbon metabolism, which break down carbon sources for usage under long-term sustained stress; thus, the fructose and glucose contents are significantly increased under high temperatures [7–10]. The KEGG analysis revealed that the DEGs were enriched in sugar metabolism, including starch and sucrose metabolism, galactose metabolism, and fructose and mannose metabolism. Further analysis of the DEGs expression profile in the sugar metabolism in response to temperature stress identified 26, 13, and 15 DEGs encoding 18, 9, and 9 enzymes involved in the starch and sucrose metabolism, galactose metabolism, and fructose and mannose metabolism, respectively. The starch, fructose, and galactose metabolic pathways were interlinked through intermediate metabolites. With the exception of a few enzymes encoded by two or four genes, most enzymes were encoded by single genes (Fig 5). Trehalose and sorbitol are important osmotically active compounds which stabilize proteins, cell walls, and membranes against stress [7–9]. Six and five genes were significantly upregulated in trehalose synthesis in the starch metabolic pathway under low- and high-temperature stress, respectively. In addition, eight of 13 genes involved in fructose and mannose metabolism were significantly downregulated under high-temperature stress. Interestingly, *SOU1* (Ao3042_05291), encoding aldehyde reductase which catalyzes the reduction of an aldose to the corresponding sugar alcohol (particularly glucose) to sorbitol [28], was significantly upregulated under low-temperature stress. Furthermore, two genes, encoding α-galactosidase which is known to catalyze the hydrolysis of the terminal α-linked galactoside residues from melibiitol to generate sorbitol [29], were upregulated under low-temperature stress. In contrast, *galM*, *galK*, and *galT* that involved in galactose catabolism, and *UDP2* and *otsA* involved in glucose metabolism were significantly downregulated under high-temperature stress (Fig 5). Gene expression profiling for the DEGs in these pathways revealed that genes in trehalose and sorbitol synthesis were upregulated in response to low-temperature stress, implying that trehalose and sorbitol contents were increased in *A*. *oryzae* cells in response to low-temperature. However, high temperatures inhibited the expression of genes in fructose, galactose, and glucose metabolism but induced trehalose synthesis, suggesting an increased accumulation of trehalose, galactose, and glucose in the *A*. *oryzae* cells in response to high-temperature stress. The detailed expression levels of the DEGs in sugar metabolic pathways are shown in S5 Table.

**Fig 6:**
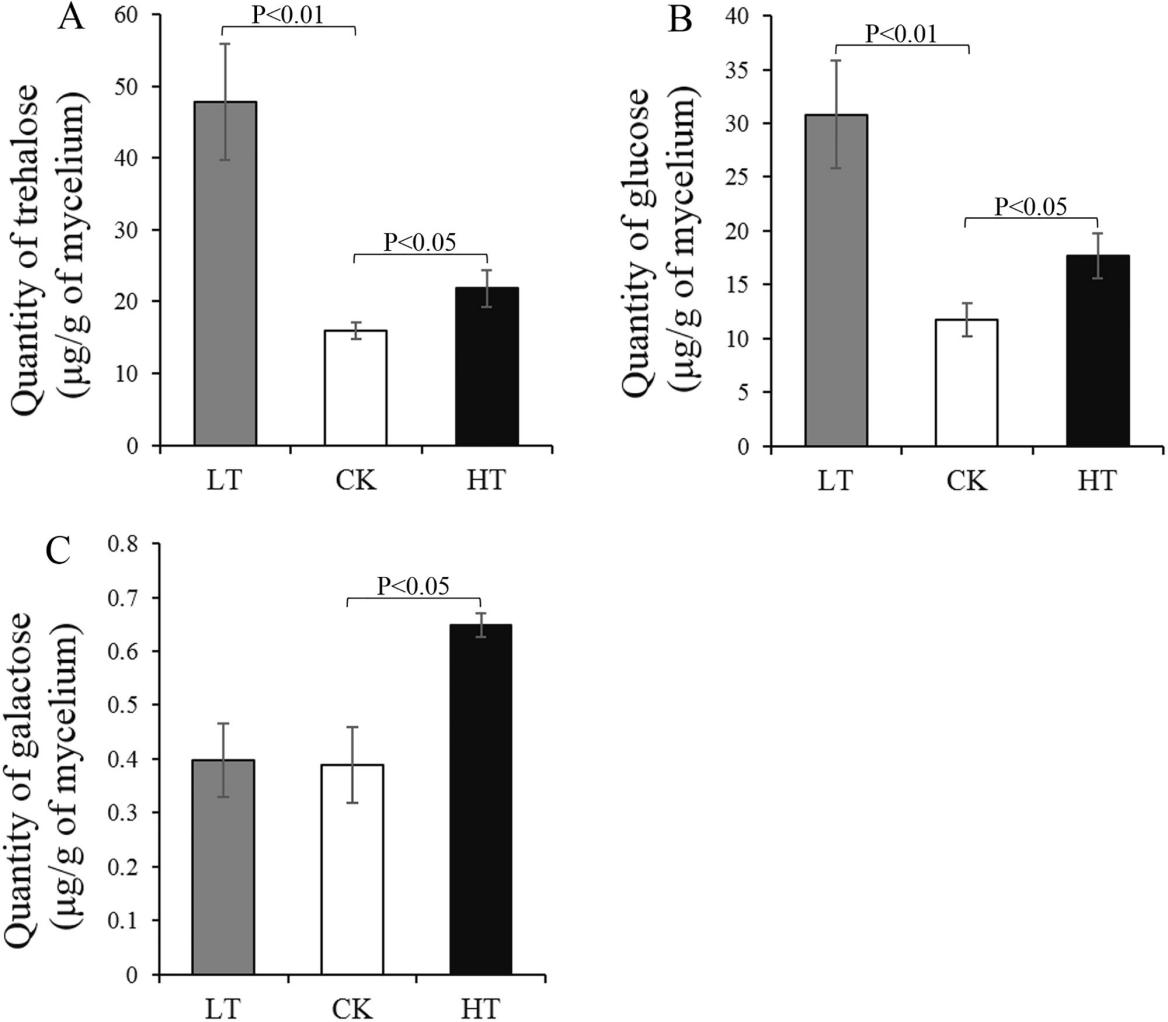
Intracellular sugar concentrations in *A*. *oryzae* under different temperature stress. The bars represent the average (± SE) of three replicates per treatment. LT, samples incubated at 22°C. CK, samples incubated at 30°C. HT, samples incubated at 42°C.

**Fig 7:**
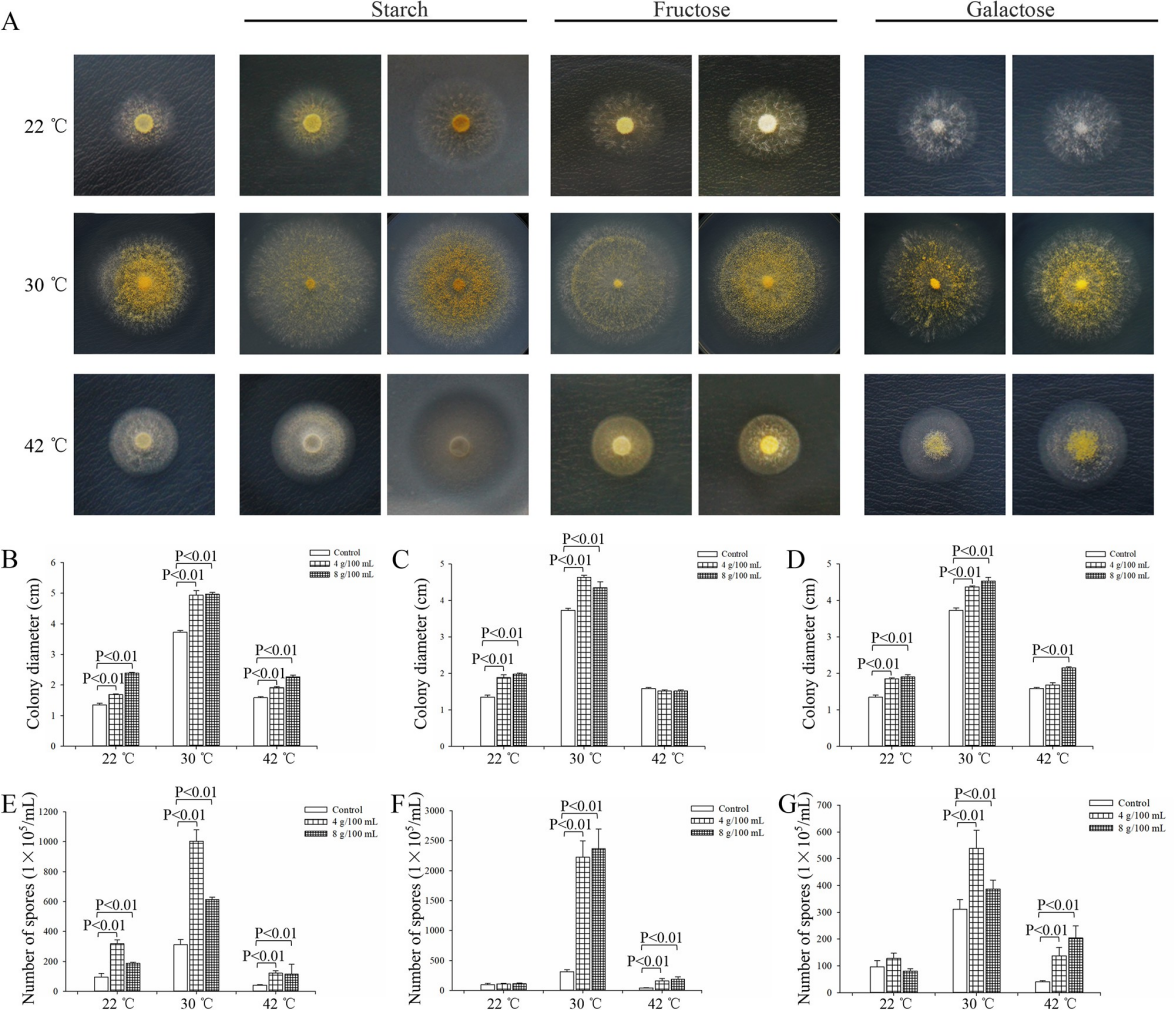
Effects of exogenous sugar on the growth of *A*. *oryzae* under low and high-temperature stress. (A) Phenotypes of *A*. *oryzae* cultured on CD medium supplemented with 4 and 8 g/100 mL of starch, fructose, and galactose or 96 h. (B-D) The colony sizes of *A*. *oryzae* cultured on CD media supplemented with starch, fructose, or galactose, respectively. (E-G) The spore density of *A*. *oryzae* cultured on CD media supplemented with starch, fructose, or galactose, respectively. CD medium with no sugar was used as the control. The values represent the average of three replicates ± SD, at a *P*<0.01 level of significance.

Further analysis revealed that the intracellular sugar contents, including trehalose and glucose, increased under low-temperature stress, while trehalose, galactose, and glucose increased under high-temperature stress (Fig 6). The trehalose content at low temperature was three times more than that of the control. Unfortunately, the contents of intracellular sorbitol and fructose were too low to be detected. To further investigate the role of sugar in temperature stress, the *A*. *oryzae* spore density and colony size in the presence/absence of starch, fructose, or galactose were studied. A concentration of 4 or 8 g/100 mL of exogenous sugar promoted the growth of *A*. *oryzae* mycelia and the formation of spores at a normal temperature of 30°C (Fig 7A). In addition, 8 g/100 mL of exogenous sugar increased the colony size of *A*. *oryzae* compared to the control under low-temperature stress (Fig 7B–7D). Besides, the ratio of colony diameter in the presence and absence of sugar was significantly higher under low temperature than at 30°C (S4A–S4C Fig). In contrast, the formation and development of *A*. *oryzae* spores were enhanced following the consumption of exogenous starch and galactose under high-temperature stress (Fig 7E and 7G; S4D and S4F Fig). The ratio of spore number in the presence of 8 g/100 mL and absence of galactose at high temperature was four times higher than at 30°C (S4F Fig). These results suggested that *A*. *oryzae* could increase the application of starch, fructose, and galactose to respond to low-temperature stress, and increase the accumulation of trehalose, glucose, and galactose to adapt to high-temperature stress.

#### DEGs involved in glycerolipid and glycerophospholipid metabolism in response to high-temperature stress

**Fig 8:**
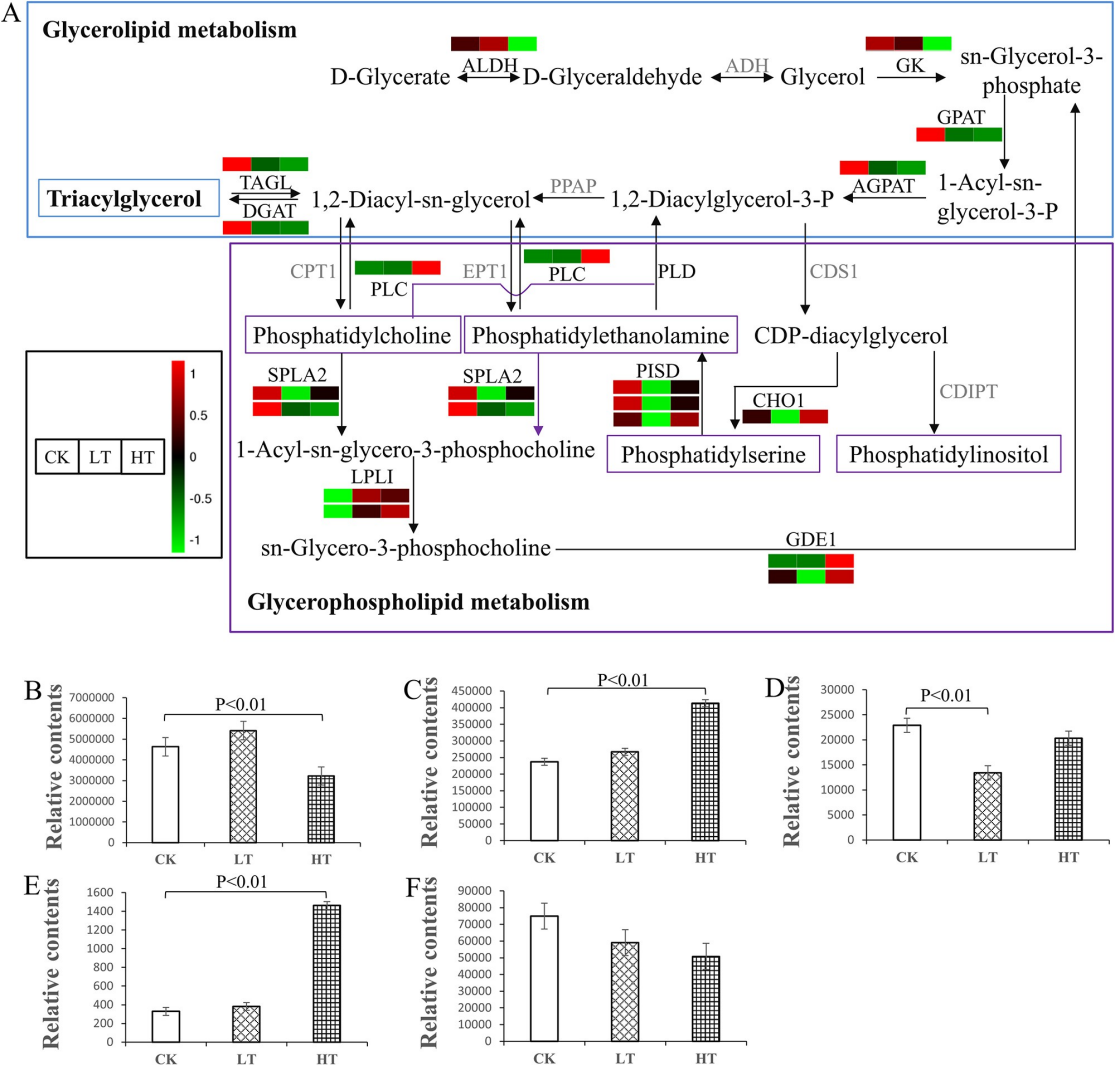
The KEGG pathways for glycerolipid and glycerophospholipid metabolism and the relative contents of their metabolites. (A) Key enzymes encoded by the DEGs in the glycerolipid and glycerophospholipid metabolism pathways. Red and green indicate the genes were upregulated and downregulated, respectively, while grey indicates no significant differences in the pathways. ALDH, aldehyde dehydrogenase. ADH, alcohol dehydrogenase. GK, glycerol kinase. GPAT, glycerol-3-phosphate O-acyltransferase. AGPAT, 1-acylglycerol-3-phosphate acyltransferase. PPAP, phosphatic acid phosphatase. DGAT, diacylglycerol O-acyltransferase 1. TAGL, triacylglycerol lipase. CPT1, diacylglycerol cholinephosphotransferase. EPT1, ethanolaminephosphotransferase. PLC, phospholipase C. PLD, phospholipase D. CDS1, phosphatidate cytidylyltransferase. CDIPT, CDP-diacylglycerol—inositol 3-phosphatidyltransferase. CHO1, CDP-diacylglycerol—serine O-phosphatidyltransferase. PISD, phosphatidylserine decarboxylase. SPLA2, secretory phospholipase A2. LPLI, lysophospholipase I. GDE1, glycerophosphodiester phosphodiesterase. Relative content of (B) triacylglycerol, (C) phosphatidylcholine, (D) phosphatidylethanolamine, (E) phosphatidylserine, and (F) phosphatidylinositol under low- or high-temperature stress. *P*<0.01 was considered the level of statistical significance. LT, samples incubated at 22°C. CK, samples incubated at 30°C. HT, samples incubated at 42°C.

A transient expression of stress-protective genes involved in lipid metabolism is triggered to defend against temperature stress. For example, the composition of cellular lipids, including glycerophospholipid PC, PE, PI, and PS, is changed, which serves as temperature sensors that sense temperature changes to initiate crucial cellular responses and developmental programs [5, 6]. The KEGG analysis of the DEGs revealed that the glycerolipid and glycerophospholipid metabolism pathways involved in response to high-temperature stress were enriched. A total of 21 and six DEGs encoding 15 and six enzymes involved in glycerophospholipid and glycerolipid metabolism were identified, respectively. With the exception of a few enzymes encoded by two or three genes, most enzymes were encoded by single genes (Fig 8A). The gene expression profile analysis of the enzymes involved in the glycerolipid metabolism pathway revealed that all of the DEGs were downregulated in response to high-temperature stress, which suggested that *A*. *oryzae* cells reduced its glycerolipid synthesis in response to high-temperature stress. In addition, the content of triacylglycerols (TGs) was significantly decreased under high-temperature stress, although its content did not differ remarkably at low temperatures (Fig 8B). Although most DEGs in the glycerophospholipid pathway were downregulated under high-temperature stress, the relative contents of PC and PS were increased (Fig 8C and 8E). In contrast, the relative contents of PE and PI were reduced under low-temperature stress (Fig 8D and 8F). Interestingly, *CHO1* was upregulated under high-temperature stress, followed by an increase in the content of PS, which was five times higher than in CK (Fig 8E). More details of the DEGs in glycerophospholipid and glycerolipid metabolism pathways are shown in S6 Table.

### Expression analysis of linoleic acid metabolic genes under low-temperature stress

**Fig 9:**
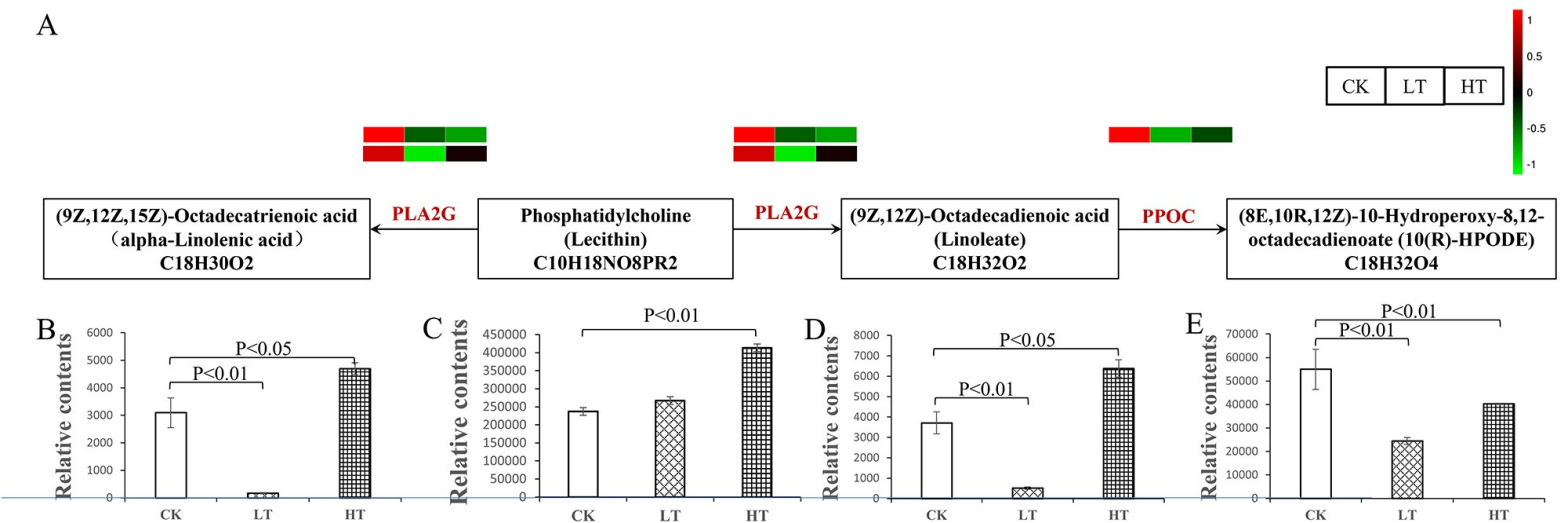
DEGs involved in the linoleic acid biosynthetic pathways, and the metabolite changes in response to temperature stress. (A) The expression pattern of the DEGs involved in the linoleic acid biosynthetic pathways. The changes in (B) alpha-linolenic acid (C_18:3_), (C) lecithin, (D) linoleate (C_18:2_), and (E) 10(R)-HPODE (C_18:2_) in response to temperature stress. The bars represent the average (± SD.) of three biological replicates. *P*<0.01 and *P*<0.05 were considered levels of statistical significance. PLA2G, phospholipase A2. PPOC, linoleate 10R-lipoxygenase. DEGs, differentially expressed genes. LT, samples incubated at 22°C. CK, samples incubated at 30°C. HT, samples incubated at 42°C.

Four DEGs involved in linoleic acid metabolism were downregulated under low-temperature stress (Fig 9A). Phospholipase A2 hydrolyzes the fatty acid from the *sn-2* position of phosphatidylcholine to generate α-linolenic acid (C_18:3_) or linoleate (C_18:2_) in linoleic acid metabolism [30]. Two genes encoding phospholipase A2 in α-linolenic acid or linoleate biosynthesis were significantly downregulated under low- or high-temperature stress. Similarly, the *PPOC* gene encoding linoleate 10R-lipoxygenase involved in the biosynthesis of 10-hydroperoxy-8,12-octadecadienoate (10(R)-HPODE) (C_18:2_) was significantly downregulated under low-temperature stress. The detailed expression levels of the DEGs in the linoleic acid pathway are shown in S7 Table. The relative contents of α-linolenic acid, linoleate, and 10(R)-HPODE decreased significantly under low-temperature stress (Fig 9B, 9D and 9E). Interestingly, although the gene expression was inhibited, the relative contents of α-linolenic acid, phosphatidylcholine, and linoleate were increased under high-temperature stress (Fig 9B–9D), while the relative contents of 10(R)-HPODE were significantly decreased (Fig 9E). Consistent with the levels of intracellular α-linolenic acid, linoleate, and 10(R)-HPODE under low-temperature stress, the expression of *PLA2G* and *PPOC* were decreased corresponding to the production of metabolites, indicating a close correlation between the physiological metabolism and transcriptional regulation. These results suggested that the downregulation of *PLA2G* and *PPOC* and the reduction of α-linolenic acid, linoleate, and 10(R)-HPODE could be one of the mechanisms that *A*. *oryzae* uses to adapt to low-temperature stress.

## Discussion

In the industrial fermentation of sake, miso, and soy sauce, temperature is the most important environmental factor that affects the growth and activity of *A*. *oryzae* [2, 3]. The involved fungus *A*. *oryzae* constantly exposed to different temperature stresses during fermentation. In this study, we conducted a preliminary study to define the effect of different temperatures on the growth of *A*. *oryzae*. The results revealed that the growth of *A*. *oryzae* mycelia and the formation of conidia were significantly inhibited under low- and high-temperature stresses, and *A*. *oryzae* barely grew below 22°C and above 42°C. Since low- and high-temperature environments prolong the process of substrate catabolism and biosynthesis owing to the decreased in cell growth and enzyme performance, the breeding of stress-tolerant strains for industrial production is highly desirable. Acclimate to temperature stress involves a transient induction of transcription that activates the genes that protect against stress [14, 31]. To identify the target genes to improve the tolerance to extreme temperatures, the genome-wide response of *A*. *oryzae* under low- and high-temperature stress was analyzed using RNA-Seq. In addition, the intracellular sugar contents and the relative contents of lipid were also determined. The findings in this study provide a theoretical basis to further understand the mechanism of *A*. *oryzae* in response to low- and high-temperature stress and lay the foundation for the molecular breeding of new *A*. *oryzae* strains that are resistant to low or high temperatures.

When exposed to extreme conditions, many microorganisms synthesize osmotically active compounds in response to environmental stresses [7–9]. For example, trehalose protects against several environmental stresses, including temperature stress [7]. In many organisms, trehalose synthesis is induced in response to specific environmental conditions, such as nutrient starvation, desiccation, changes in osmolarity, oxidative stress, and exposure to mild heat shock [32, 33]. Under normal physiological conditions, the intracellular trehalose in fungus is stable but typically changes when the cells are under osmotic stress [34, 35]. Trehalose enhances *Volvariella volvacea* tolerance to low-temperature stress, and the high content of trehalose in *V*. *volvacea* strain VH3 contributes to its high tolerance to low-temperature stress than strain V23 [11]. Transcriptome analysis and quantification of intracellular sugar revealed that the *amyA*, *MGAM*, and *treT* involved in trehalose synthesis were upregulated, corresponding to the accumulation of intracellular trehalose under low-temperature stress. Chi et al. demonstrate that corn starch can be used as the substrate for cell growth and trehalose accumulation in *Saccharomycopsis fibuligera* [36]. In our study, exogenous starch enhanced the tolerance of *A*. *oryzae* to low-temperature stress, implying that *A*. *oryzae* might increase the expression levels of *amyA*, *MGAM*, and *treT* to increase the utilization of the exogenous starch to accumulate trehalose, enhancing the adaptation to low-temperature stress. These results demonstrate that trehalose biosynthesis plays a positive role in enhancing *A*. *oryzae* tolerance against low-temperature stress.

Zhao *et al* [11] demonstrated that various osmotic regulatory materials, such as sugar and sugar alcohol, accumulated in *Lentinula edodes* mycelia under high-temperature stress. At high temperature, galactose acts as a penetrating agent to stabilize the membrane structure and protect the stability of proteins [7]. In addition, high temperature significantly promote the accumulation of galactose, fructose, and glucose to enhance heat resistance [37]. Our results demonstrated that galactose and glucose metabolism were inhibited in *A*. *oryzae* through the downregulation of *galM*, *galK*, *galT*, *UDP2*, and *otsA*, accompanied by the accumulation of intracellular galactose and glucose under high-temperature stress. In addition, the DEGs involved in starch metabolism were upregulated under high-temperature stress, followed by an increased accumulation of intracellular glucose. Furthermore, the exogenous galactose significantly promoted mycelia growth and the formation of spores at high temperature, which also supported that *A*. *oryzae* utilize exogenous galactose to increase the accumulation of intracellular galactose, enhancing its resistance to high-temperature stress. Trehalose is widely recognized as a protective sugar that protects cells from stress damage and is considered as a carbon storage source to provide energy under long-term sustained stress in fungi [35]. In thermophilic fungi, the level of trehalose is increased under heat shock [10]. Our results revealed that the upregulation of *treT* gene in the trehalose synthesis was accompanied by the accumulation of trehalose at high temperature. In addition, cells suffer heat-induced oxidative stress at high temperature [38]. Interestingly, polyols, known as sugar alcohols, stabilize macromolecules, effectively scavenging hydroxyl radicals, which prevents oxidative damage to membranes and enzymes. Therefore, the accumulation of galactose, glucose, and trehalose could decrease the damage from oxidative stress induced by high temperature and improve the fitness of *A*. *oryzae*.

The exposure of *A*. *oryzae* cells to low or high temperature potentially destroys the integrity of the cell membrane, thus, altering the plasma membrane function. These changes can include the loss of selective permeability, which increases fluidity and decreases permeability. The cell membrane is the first target that suffers from the injurious effects of stress; hence, the cellular lipid profiles are modified to adapt to temperature stress [5, 10, 39, 40]. Lipids serve as temperature sensors that sense temperature changes to initiate crucial cellular responses and developmental programs [6]. Here we discussed the roles of linoleic acid, glycerolipid, and glycerophospholipid in response to low- or high-temperature stress in *A*. *oryzae*.

Temperature directly affects the composition of fatty acids (FAs), including unsaturated fatty acids (UFAs), which are increased as the temperature decreases [41], although some reports have found opposite results. For example, when the growth temperature of *Trichoderma reesei* was reduced below 20~26°C, the content of UFAs and linolenic acid in the mycelia was significantly decreased [40]. In contrast, UFAs were nearly temperature-independent in *Neurospora crassa*, although the contents of palmitic and linoleic acid were decreased when the temperature was lowered to between 20 and 26°C [40]. In addition, Ahumada-Rudolph *et al*. [42] observed no significant differences between saturated FAs and UFAs in *Epicoccum nigrum* at 6 and 25°C. Consistent with these research, *PLA2G* and *PPOC* involved in the biosynthesis of octadecatrienoic acid (C_18:3_), octadecadienoic acid (C_18:2_), and hydroperoxy-8,12-octadecadienoate (C_18:3_) were significantly downregulated under low-temperature stress, significantly decreasing the content of these FAs. This indicated that the content of linoleic acid in *A*. *oryzae* is decreased in response to low-temperature stress.

In contrast, the lipid profile analysis revealed that high temperature promoted the accumulation of octadecatrienoic acid and octadecadienoic acid, which demonstrated that the contents of UFAs (linolenic acid) in *A*. *oryzae* are increased to maintain membrane stability under high-temperature stress. Indeed, the contents of UFAs play an essential role in maintaining suitable bilayer flexibility of the membranes; thus, an increased UFAs concentration is an essential strategy to maintain the membranes in an appropriate fluid state at high temperature [43]. The is consistent with the findings in this study, where the accumulation of linoleic acid was increased in *A*. *oryzae* cells in response to high temperature stress.

Glycerophospholipid is also crucial in response to stress in thermophilic and psychrophilic yeasts. The primary glycerophospholipids include PC, PE, PI, PS, and PA, which are essential structural components of membranes, with PI, PE, and PC playing an important role in response to temperature. The contrasting trends of PI and PE abundances maintain the membrane properties in a physiologically optimal state. The small headgroup and the unsaturated hydrocarbon chains of PE form a conical shape, creating the inverted hexagonal (H_II_) phase in PE membranes [44]. In contrast, PI has an inositol phosphate headgroup, which forms intermolecular hydrogen bonds that cause the bilayer condensation [45]. Thus, there is a possibility for the adaptation of biological membranes to temperature changes by altering PC, PI, and PE contents in mitochondrial and endoplasmic reticular membranes. For example, the content of PI is increased in psychrophilic yeasts, while the content of PE is decreased with an increase in the cultivation temperature (24°C and 37°C) [5]. Besides, PE is decreased in *S*. *cerevisiae*, while PI is more abundant to adapt to increasingly high temperature. In contrast, changes in the abundance of PC do not result in a significant temperature-dependent trend [46, 47]. Herein, most of the DEGs involved in the glycerophospholipid metabolism were downregulated, followed by a decrease in the contents of PE and PI under low-temperature stress in *A*. *oryzae*. In addition, the PC and PS levels were significantly increased under high-temperature stress. These results demonstrate that the levels of PE and PI are decreased in *A*. *oryzae* to adapt to low-temperature stress, while PC and PS contents are increased to respond to high-temperature stress. Thus, an interchange among PE, PI, PC, and PS levels might provide an efficient way to fine-tune the membrane properties at varying temperatures in *A*. *oryzae*.

TGs are the major type of acyl lipid produced by glycerolipids in fungi [48]. The accumulation of TGs may function in increasing fungal reproduction, creating lower cellular toxicity, or simply partitioning abundant TGs to store energy rather than relying on membrane fluidity [49]. Although TGs are increased at high and low temperature, the two conditions have different effects. Heat stress in fungi initiates the denaturation, aggregation, or misfolding of proteins during translation, which leads to irreversible cellular damage [50]. In addition, heat stress decreases the rates of biochemical reactions and enzymes activities. Unlike heat stress, the effects of cold stress are reversible when the organism is brought closer to optimal temperature if ice formation has not permanently damaged the cell [51, 52]. Herein, gene expression and metabolite analyses revealed that TGs contents in *A*. *oryzae* are decreased under high-temperature stress and increased them under low-temperature stress to avoid damage to the cells from low temperature. However, the changes in TG concentrations under temperature stress in *A*. *oryzae* remain unclear and warrant further investigation.

## Conclusions

This paper focused on the transcriptomic profiles, intracellular sugar contents, and lipid metabolomics in *A*. *oryzae* under different temperature stresses and explored the underlying response mechanism of *A*. *oryzae* to low- and high-temperature stresses. Temperature mainly affects the sugar metabolism, glycerolipid pathway, glycerophospholipid pathway, and linoleic acid metabolism in *A*. *oryzae*. Under low-temperature stress, the genes involved in trehalose synthesis and starch metabolism are upregulated, leading to a corresponding intracellular accumulation of trehalose. However, under high-temperature stress, the genes involved in fructose, galactose, and glucose metabolism are inhibited, leading to an increased accumulation of intracellular galactose and glucose, which enhances the adaptability of *A*. *oryzae* to high temperature and decreases oxidative stress-related damage induced by exposure to high temperature. In addition, high temperature hinders the normal flow of TG pathway and reduces the accumulation of TG products. Furthermore, interchange levels of PE, PI, PC, and PS metabolism is one of the molecular mechanisms in response to varying temperatures in *A*. *oryzae*. The findings in this study provide a theoretical basis for understanding the response mechanism of *A*. *oryzae* to low and high temperatures, which can be used to screen for low/high-temperature-resistant strains suitable for industrial applications. It also lays a foundation for the molecular breeding of new low/high-temperature-resistant *A*. *oryzae* strains.

## Supporting information

S1 Fig Significantly enriched GO terms associated with upregulated or downregulated genes identified in the LT-vs-CK group. (TIF)

S2 Fig Significantly enriched GO terms associated with upregulated or downregulated genes identified in the HT-vs-CK group. (TIF)

S3 Fig The correlation between the results of qRT-PCR and those DEGs selected from RNA-seq. (TIF)

S4 Fig Compared the ratio of colony diameter and spore number in the presence and absence of starch, fructose, or galactose under low- and high-temperature stresses. (A-C) Ratio of colony diameter in the presence and absence of starch, or fructose, or galactose. (D-F) Ratio of spore number in the presence and absence of starch, or fructose, or galactose. 4%/without, Ratio of colony diameter (or spore number) with 4% starch, or fructose, or galactose and without additional sugar. 8%/without, Ratio of colony diameter (or spore number) with 8% starch, or fructose, or galactose and without additional sugar.
(TIF)

S1 Table The qRT-PCR primers of the DEGs in response to temperature stress. (DOCX)

S2 Table The details of 265 co-expressed DEGs in three temperature treatments. (XLSX)

S3 Table The details of the DEGs in the GO term of response to stimulus in the LT-vs-CK group. (DOCX)

S4 Table The details of the DEGs in the GO term of response to stimulus in HT-vs-CK. (DOCX)

S5 Table The expression levels of the DEGs in sugar mechanisms. (DOCX)

S6 Table The expression levels of the DEGs in the metabolic pathways of glycerophospholipids and glycerolipids. (DOCX)

S7 Table The expression levels of the DEGs in the metabolic pathway of linoleic acid. (DOCX)
