# Supplementary figures and images for: Transcriptomic analysis reveals Aspergillus oryzae responds to temperature stress by regulating sugar metabolism and lipid metabolism
Source: PLoS One. 2022 Sep 12;17(9):e0274394. doi: 10.1371/journal.pone.0274394 (PMC9467314; doi:10.1371/journal.pone.0274394)

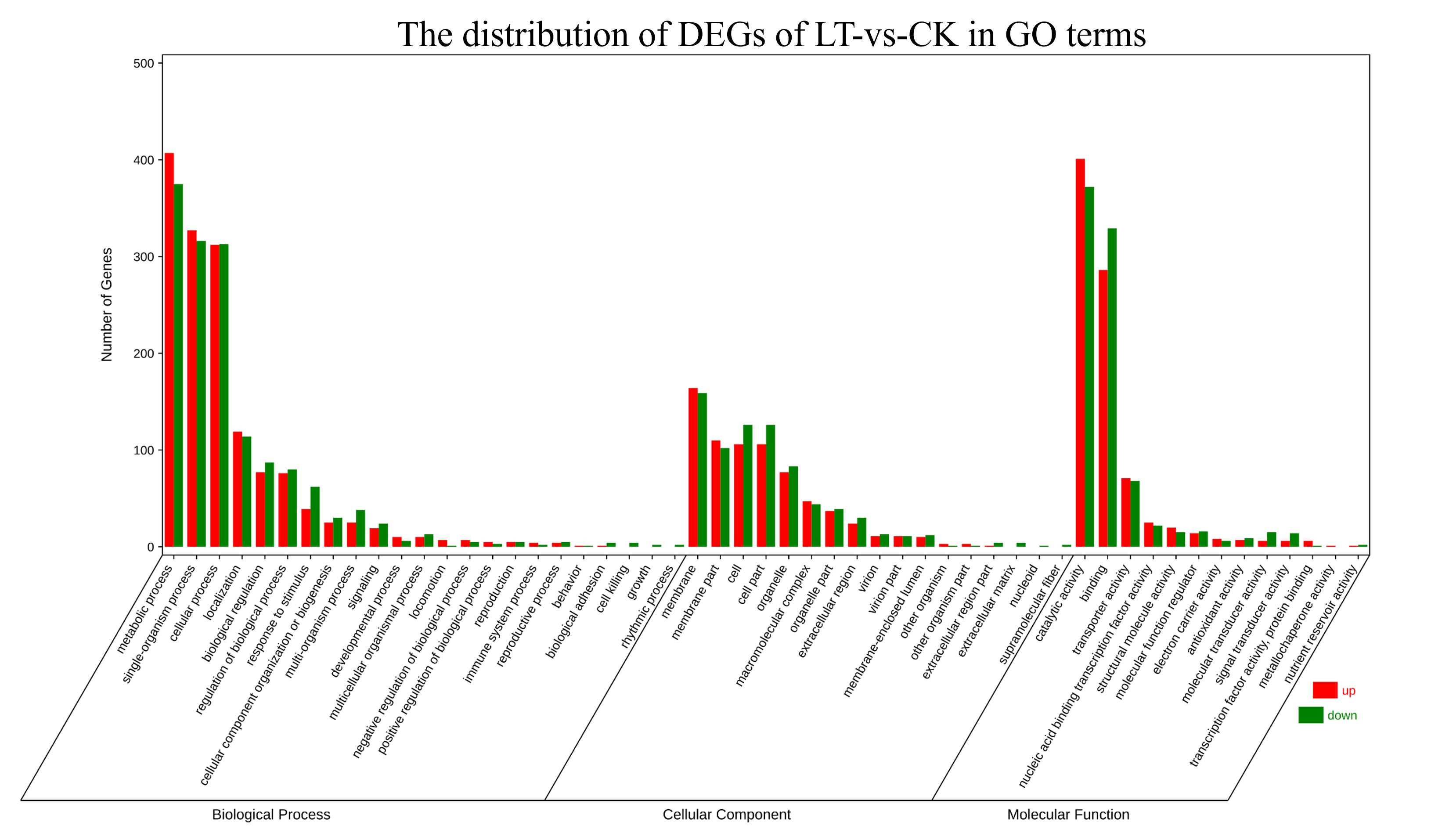

Supplement: S1 Fig — (TIF) [file pone.0274394.s001.tif]

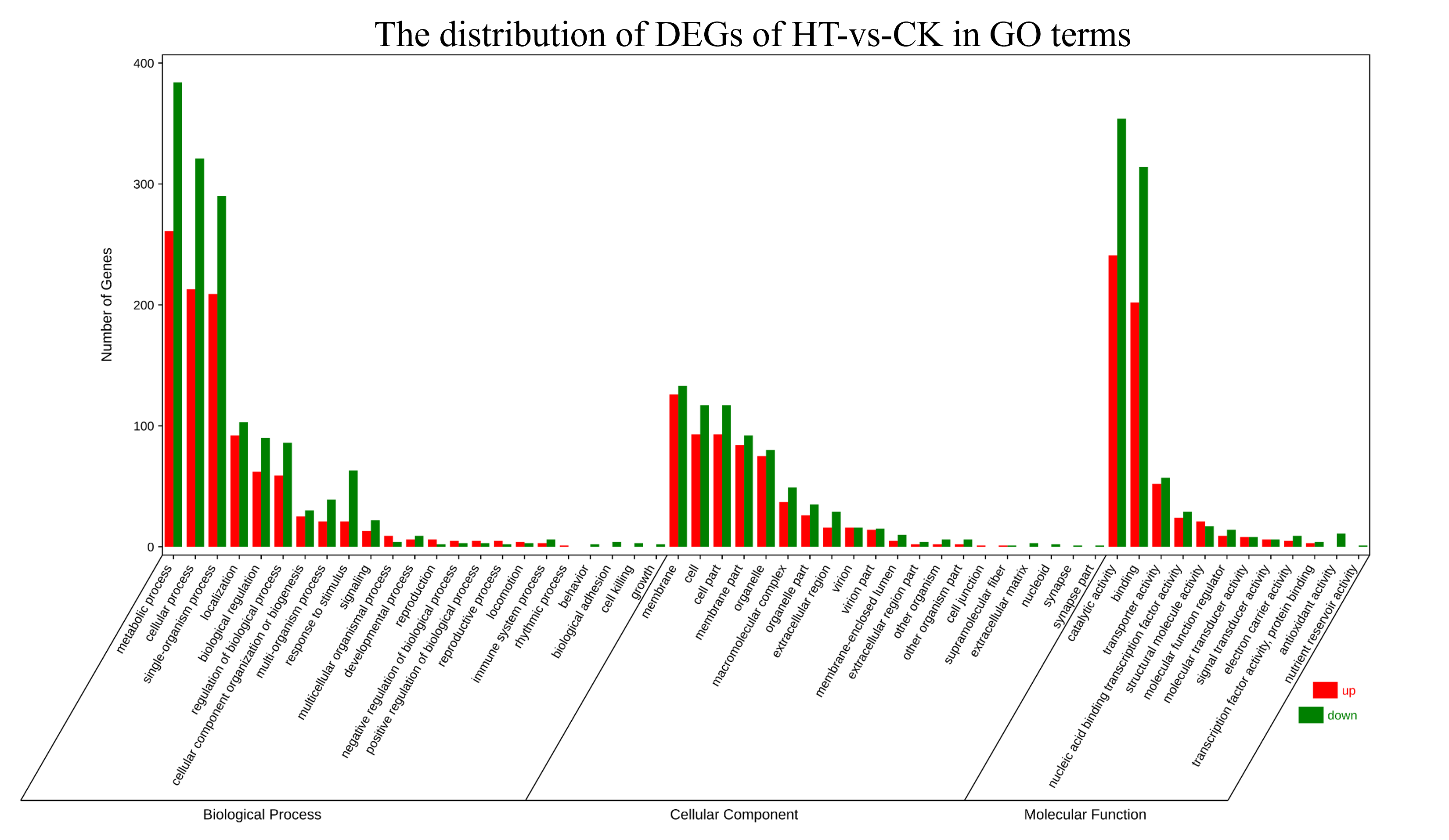

Supplement: S2 Fig — (TIF) [file pone.0274394.s002.tif]

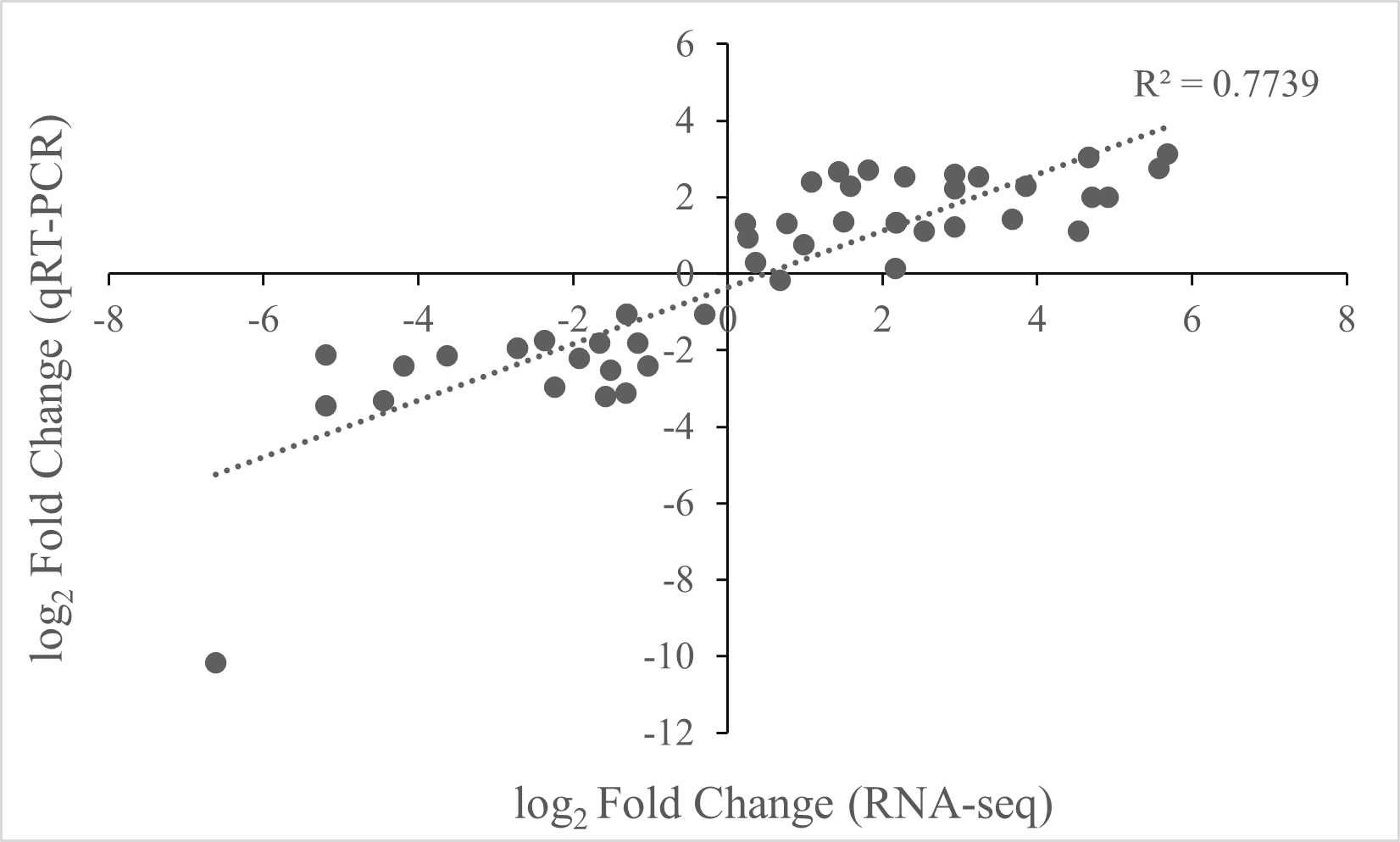

Supplement: S3 Fig — (TIF) [file pone.0274394.s003.tif]

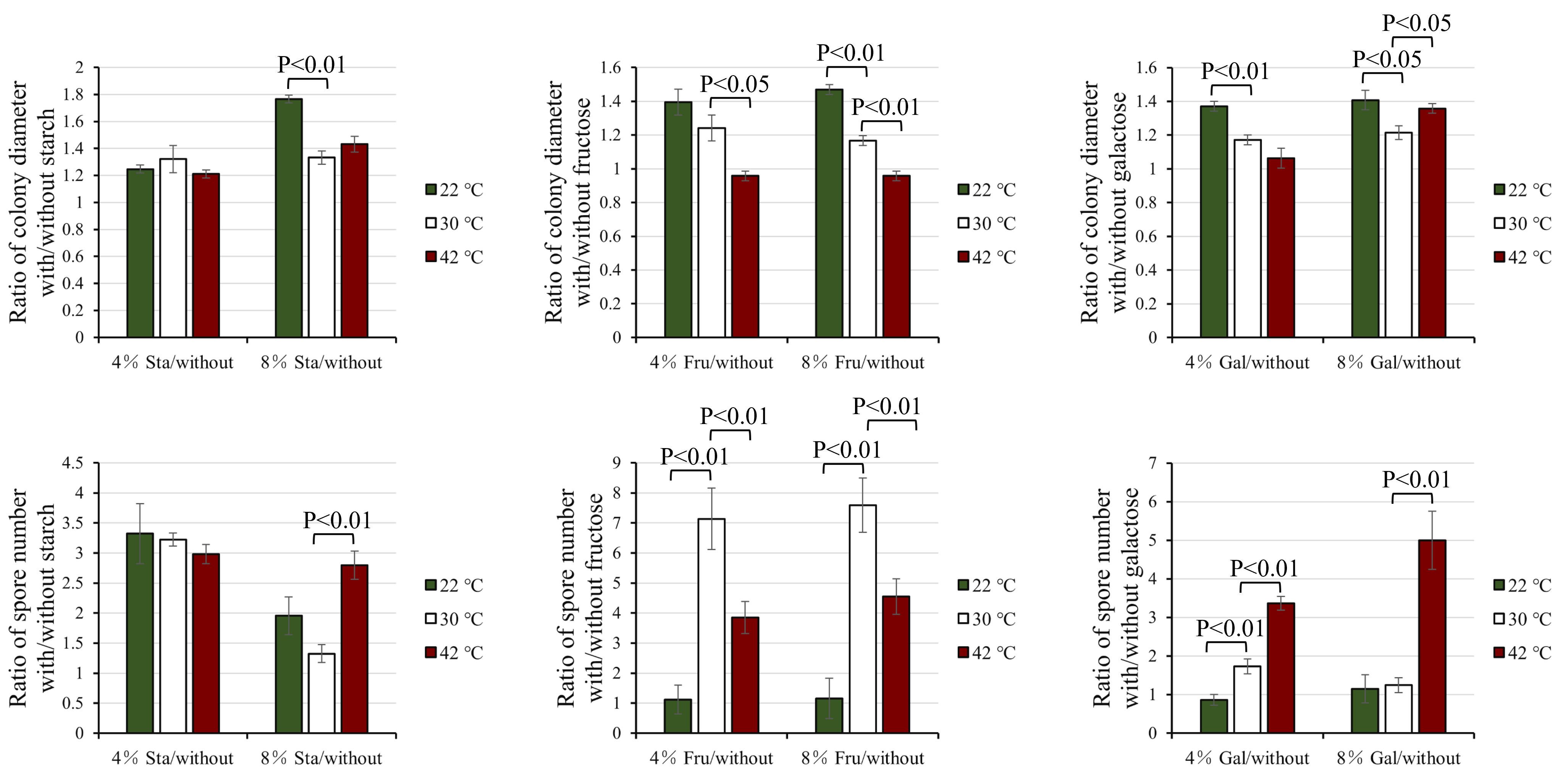

Supplement: S4 Fig — (A-C) Ratio of colony diameter in the presence and absence of starch, or fructose, or galactose. (D-F) Ratio of spore number in the presence and absence of starch, or fructose, or galactose. 4%/without, Ratio of colony diameter (or spore number) with 4% starch, or fructose, or galactose and without additional sugar. 8%/without, Ratio of colony diameter (or spore number) with 8% starch, or fructose, or galactose and without additional sugar. (TIF) [file pone.0274394.s004.tif]
