# Supplementary material for: Transcriptomic analysis reveals Aspergillus oryzae responds to temperature stress by regulating sugar metabolism and lipid metabolism
Source: PLoS One. 2022 Sep 12;17(9):e0274394. doi: 10.1371/journal.pone.0274394 (PMC9467314; doi:10.1371/journal.pone.0274394)
Supplement: S1 Table — (DOCX) [file pone.0274394.s005.docx]

| **S1 Table qRT-PCR primers of DEGs in response to temperature stress** | |
| --- | --- |
| Gene IDs | Primer sequence 5'→3' |
| Ao3042_07786 | F: AAGTCCACCGCTCTGCTTAC; R: GCTTGTATCCGAAAGGGGCT |
| Ao3042_06209 | F: TGCGTGGTATCTTCTCCGAC; R: TCGTACACTATCCACGTCGC |
| Ao3042_10549 | F: CGACTCTTATCACGGTGCTGC; R: CGGAGTGTCCTTGATAGGGTTT |
| Ao3042_02707 | F: CCACTCACCGTCAGAGATCG; R: AGCTCCCGAACTAGACCCTT |
| Ao3042_05018 | F: TGTAGCAGGGCGAGTCAAAG; R: AGATAAGGCGAACCGCCAAA |
| Ao3042_10146 | F: GCGCGGAAAATGGATCACTC; R: TGAGTCCTCGTAGATGCCGA |
| Ao3042_09236 | F: CCTAATCCGCACCTCCCTAAG; R: TCAACTCCAGCCCATCACAAC |
| Ao3042_08203 | F: ACTACTGGTTACACCGTGCTTT; R: CTGGCGTACTCGGCTGTAA |
| Ao3042_08269 | F: GAAAGCAATCGGTGCAGTCC; R: CGGTTCGATGTAGGTTCCGT |
| Ao3042_05291 | F: GTCGAGGAGGCAGTCAACAA; R: CGGATCGATAGGCGTGAGAG |
| Ao3042_02662 | F: ATTGGTGCTGGTGTGGAGAG; R: CCACGGTCCTTAGCCATGTT |
| Ao3042_04763 | F: TAGCGCCAGCAGAAGTCAAA; R: GACGGACCAGCTCTTGTTCA |
| Ao3042_05624 | F: GGAGCCACGACCCAATACAT; R: TCTTGTCTTGCGGGTCAGTC |
| Ao3042_08133 | F: CAAATTTTTCCCGCCAGGCA; R: GGGTCACTTTTGCTAGGGCT |
| Ao3042_08758 | F: GAGGGTCCTCTCATCTCCGT; R: TTTCCACCATTGCCACCCTT |
| Ao3042_08961 | F: CTTTCGACGTCAGCGAGAGT; R: CGATGGAGATACCGTGACCG |
| Ao3042_07367 | F: GACGTCTCCGGTTGTTCGAT; R: GTACCAGGAACGAGCCCAAG |
| Ao3042_03902 | F: TCGCCGCACTAGAAGCTATG; R: CATGGTCGATCTCGTCAGCA |
| Ao3042_08394 | F: CGCACAGCAATACAGAACGG; R: CCACGGGATAGGAAACCTCG |
| Ao3042_10146 | F: GCGCGGAAAATGGATCACTC; R: TGAGTCCTCGTAGATGCCGA |
| RH-actin | F: GACAACATCCAGGGTATCACTAAGC; R: GGTCTCCTCGTAGATCATGGCA |
| F: Forward primer, R: Reverse primer; RH-actin: Reference gene in qRT-PCR analysis | |
