# Supplementary material for: Transcriptomic analysis reveals Aspergillus oryzae responds to temperature stress by regulating sugar metabolism and lipid metabolism
Source: PLoS One. 2022 Sep 12;17(9):e0274394. doi: 10.1371/journal.pone.0274394 (PMC9467314; doi:10.1371/journal.pone.0274394)
Supplement: S3 Table — (DOCX) [file pone.0274394.s007.docx]

| **S3 Table The details of DEGs in the GO term of response to stimulus in LT-vs-CK** | | | | | | | |  |  |
| --- | --- | --- | --- | --- | --- | --- | --- | --- | --- |
| Gene_id | LT_fpkm | CK_fpkm | log_2_FoldChange(LTvsCK) | pval | significant | biological_process | gene_description |  |  |
| Ao3042_00276 | 655.1867 | 4026.856 | -2.5331 | 0 | DOWN | GO:0050896 | hypothetical protein |  |  |
| Ao3042_00432 | 0.21259 | 25.7091 | -6.8315 | 1.85E-33 | DOWN | GO:0050896 | hypothetical protein |  |  |
| Ao3042_00613 | 76.42015 | 169.1703 | -1.0599 | 1.48E-08 | DOWN | GO:0050896 | hypothetical protein |  |  |
| Ao3042_00675 | 4.956114 | 42.63216 | -3.0181 | 3.08E-14 | DOWN | GO:0050896 | AAA+-type ATPase |  |  |
| Ao3042_00802 | 12.66059 | 4.356542 | 1.6257 | 4.29E-07 | UP | GO:0050896 | hypothetical protein |  |  |
| Ao3042_00947 | 15.45241 | 36.56744 | -1.1561 | 0.00053465 | DOWN | GO:0050896 | extracellular triacylglycerol lipase |  |  |
| Ao3042_01012 | 9.431262 | 45.64041 | -2.1882 | 6.42E-10 | DOWN | GO:0050896 | erythromycin esterase like protein |  |  |
| Ao3042_01093 | 11.72242 | 33.03924 | -1.4083 | 2.02E-05 | DOWN | GO:0050896 | protein involved in cell differentiation/sexual development |  |  |
| Ao3042_01098 | 104.1718 | 27.62125 | 2.0017 | 3.15E-19 | UP | GO:0050896 | cytosine deaminase |  |  |
| Ao3042_01138 | 13.80663 | 2.515886 | 2.5428 | 0.00014388 | UP | GO:0050896 | UDP-N-acetylmuramate-alanine ligase |  |  |
| Ao3042_01245 | 2088.847 | 837.5431 | 1.4051 | 9.19E-260 | UP | GO:0050896 | alcohol dehydrogenase class V |  |  |
| Ao3042_01327 | 30.60517 | 12.46883 | 1.382 | 0.00024606 | UP | GO:0050896 | salicylate hydroxylase |  |  |
| Ao3042_01364 | 58.71458 | 15.74237 | 1.9857 | 1.76E-11 | UP | GO:0050896 | hypothetical protein |  |  |
| Ao3042_01923 | 4.022375 | 12.88639 | -1.5931 | 0.00018262 | DOWN | GO:0050896 | signal transduction histidine kinase |  |  |
| Ao3042_02202 | 3.656331 | 18.17706 | -2.2271 | 3.58E-13 | DOWN | GO:0050896 | 3-oxoacyl-[acyl-carrier protein] reductase |  |  |
| Ao3042_03091 | 52.90375 | 4.525825 | 3.6337 | 8.94E-14 | UP | GO:0050896 | hypothetical protein |  |  |
| Ao3042_03117 | 9.353147 | 3.821737 | 1.3778 | 0.0011598 | UP | GO:0050896 | WD40 repeat protein |  |  |
| Ao3042_03332 | 8.011386 | 22.73512 | -1.4182 | 0.001747 | DOWN | GO:0050896 | RTA1 domain protein |  |  |
| Ao3042_03583 | 9.415509 | 1.586212 | 2.656 | 2.51E-05 | UP | GO:0050896 | hypothetical protein |  |  |
| Ao3042_03713 | 214.7122 | 104.924 | 1.1196 | 2.90E-11 | UP | GO:0050896 | septin family protein |  |  |
| Ao3042_04063 | 12.62104 | 34.66242 | -1.371 | 4.69E-09 | DOWN | GO:0050896 | EF-hand superfamily Ca2+-modulated protein |  |  |
| Ao3042_04131 | 56.08385 | 1091.109 | -4.1955 | 8.22E-167 | DOWN | GO:0050896 | hypothetical protein |  |  |
| Ao3042_04162 | 73.58559 | 38.07572 | 1.0371 | 6.68E-07 | UP | GO:0050896 | hypothetical protein |  |  |
| Ao3042_04450 | 312.3391 | 148.9258 | 1.1551 | 2.82E-15 | UP | GO:0050896 | septin CDC10 |  |  |
| Ao3042_04535 | 3.633093 | 15.99174 | -2.0515 | 1.16E-07 | DOWN | GO:0050896 | 5'-3' exonuclease |  |  |
| Ao3042_04764 | 20.39415 | 64.8784 | -1.583 | 1.90E-15 | DOWN | GO:0050896 | peroxisomal long-chain acyl-CoA transporter ABC superfamily |  |  |
| Ao3042_04843 | 120.0034 | 54.97853 | 1.2127 | 1.19E-09 | UP | GO:0050896 | hypothetical protein |  |  |
| Ao3042_05216 | 48.59464 | 103.5042 | -1.0042 | 4.74E-15 | DOWN | GO:0050896 | bZIP transcription factor |  |  |
| Ao3042_05274 | 26.48234 | 10.51368 | 1.4193 | 9.81E-05 | UP | GO:0050896 | Tyrosyl-tRNA synthetase |  |  |
| Ao3042_05298 | 55.46697 | 28.21334 | 1.0618 | 1.10E-05 | UP | GO:0050896 | threonine/serine dehydratase |  |  |
| Ao3042_05436 | 146.46 | 20.60947 | 2.9157 | 1.17E-58 | UP | GO:0050896 | hypothetical protein |  |  |
| Ao3042_05706 | 284.9899 | 109.0005 | 1.4732 | 6.56E-41 | UP | GO:0050896 | GTP-binding protein CRFG/NOG1 |  |  |
| Ao3042_05775 | 34.87651 | 170.0637 | -2.1992 | 8.71E-20 | DOWN | GO:0050896 | hypothetical protein |  |  |
| Ao3042_05848 | 5.322647 | 14.59388 | -1.3686 | 0.00085862 | DOWN | GO:0050896 | hypothetical protein |  |  |
| Ao3042_06040 | 42.60702 | 123.7041 | -1.4511 | 5.49E-10 | DOWN | GO:0050896 | putative Zn-finger protein |  |  |
| Ao3042_06147 | 13.69406 | 30.42883 | -1.0653 | 1.05E-08 | DOWN | GO:0050896 | bacteriophytochrome |  |  |
| Ao3042_06225 | 105.6741 | 54.53213 | 1.041 | 1.27E-08 | UP | GO:0050896 | xanthine/uracil permease |  |  |
| Ao3042_06264 | 6.336711 | 16.76774 | -1.3173 | 3.81E-06 | DOWN | GO:0050896 | DNA replication checkpoint protein CHL12/CTF18 |  |  |
| Ao3042_06402 | 8.549035 | 3.334517 | 1.4449 | 0.001328 | UP | GO:0050896 | hypothetical protein |  |  |
| Ao3042_06486 | 5.816512 | 19.5131 | -1.6596 | 6.03E-06 | DOWN | GO:0050896 | hypothetical protein |  |  |
| Ao3042_06569 | 777.4014 | 2674.939 | -1.6962 | 4.51E-207 | DOWN | GO:0050896 | hypothetical protein |  |  |
| Ao3042_06583 | 1.281905 | 82.92017 | -5.9288 | 6.96E-110 | DOWN | GO:0050896 | hypothetical protein |  |  |
| Ao3042_06644 | 0.370176 | 7.935645 | -4.3355 | 4.59E-08 | DOWN | GO:0050896 | multidrug/pheromone exporter ABC superfamily |  |  |
| Ao3042_06744 | 23.60233 | 58.13935 | -1.214 | 5.78E-11 | DOWN | GO:0050896 | peroxisomal long-chain acyl-CoA transporterABC superfamily |  |  |
| Ao3042_06761 | 24.25892 | 66.49127 | -1.3681 | 1.35E-15 | DOWN | GO:0050896 | hypothetical protein |  |  |
| Ao3042_06807 | 97.77652 | 280.0043 | -1.4313 | 4.45E-38 | DOWN | GO:0050896 | annexin | |  |
| Ao3042_06982 | 3.329342 | 21.9876 | -2.6368 | 0.0015105 | DOWN | GO:0050896 | Ras-related GTPase |  |  |
| Ao3042_07031 | 4.353261 | 20.20125 | -2.1277 | 1.06E-09 | DOWN | GO:0050896 | hypothetical protein |  |  |
| Ao3042_07095 | 170.3138 | 433.8627 | -1.2625 | 2.99E-21 | DOWN | GO:0050896 | glutathione S-transferase |  |  |
| Ao3042_07326 | 11.54512 | 47.65183 | -1.9587 | 1.21E-16 | DOWN | GO:0050896 | AAA+-type ATPase |  |  |
| Ao3042_07373 | 24.84504 | 61.8344 | -1.2289 | 1.81E-06 | DOWN | GO:0050896 | AAA+-type ATPase |  |  |
| Ao3042_07497 | 138.5072 | 20.58268 | 2.837 | 4.57E-50 | UP | GO:0050896 | hypothetical protein |  |  |
| Ao3042_07653 | 29.25415 | 105.745 | -1.7673 | 1.49E-05 | DOWN | GO:0050896 | hypothetical protein |  |  |
| Ao3042_07654 | 10.42673 | 43.42998 | -1.9718 | 2.12E-17 | DOWN | GO:0050896 | signal transduction histidine kinase |  |  |
| Ao3042_07715 | 23.04285 | 9.17893 | 1.4145 | 1.82E-05 | UP | GO:0050896 | hypothetical protein |  |  |
| Ao3042_07891 | 39.73278 | 15.25462 | 1.4677 | 0.0015468 | UP | GO:0050896 | threonine dehydrogenase |  |  |
| Ao3042_07926 | 0.933974 | 15.04577 | -3.9232 | 0.00028482 | DOWN | GO:0050896 | hypothetical protein |  |  |
| Ao3042_08089 | 10.95161 | 1.041589 | 3.4809 | 8.97E-07 | UP | GO:0050896 | dynamin family GTPase putative |  |  |
| Ao3042_08157 | 136.9557 | 42.94886 | 1.7596 | 1.17E-27 | UP | GO:0050896 | catalase | |  |
| Ao3042_08217 | 12.81742 | 32.22015 | -1.2433 | 1.70E-05 | DOWN | GO:0050896 | hypothetical protein |  |  |
| Ao3042_08274 | 0 | 20.55575 | -4.5298 | 0.00045094 | DOWN | GO:0050896 | hypothetical protein |  |  |
| Ao3042_08349 | 10.91101 | 54.18212 | -2.2254 | 3.77E-27 | DOWN | GO:0050896 | HAMP domain protein |  |  |
| Ao3042_08362 | 539.9574 | 228.4209 | 1.3277 | 2.91E-32 | UP | GO:0050896 | cytochrome c oxidase subunit IV/COX5b |  |  |
| Ao3042_08612 | 28.35177 | 750.2865 | -4.6393 | 0 | DOWN | GO:0050896 | vacuolar sorting protein VPS1 dynamin |  |  |
| Ao3042_08640 | 8.647695 | 22.83307 | -1.3142 | 0.00022571 | DOWN | GO:0050896 | sensory transduction histidine kinase putative |  |  |
| Ao3042_08713 | 4.2596 | 31.42282 | -2.7964 | 1.20E-06 | DOWN | GO:0050896 | putative panthothenate kinase/uridine kinase-related protein |  |  |
| Ao3042_08724 | 33.54983 | 17.21884 | 1.0489 | 0.00078023 | UP | GO:0050896 | dihydroxy-acid dehydratase |  |  |
| Ao3042_08847 | 13.08977 | 30.26051 | -1.1224 | 6.19E-08 | DOWN | GO:0050896 | PAS/PAC domain protein |  |  |
| Ao3042_08901 | 8.230237 | 39.06823 | -2.1604 | 3.05E-18 | DOWN | GO:0050896 | hypothetical protein |  |  |
| Ao3042_09055 | 8.401786 | 18.88615 | -1.082 | 0.0011747 | DOWN | GO:0050896 | RNA Helicase |  |  |
| Ao3042_09183 | 348.7646 | 132.038 | 1.4879 | 4.24E-49 | UP | GO:0050896 | catalase | |  |
| Ao3042_09234 | 6.935289 | 32.0359 | -2.1211 | 8.06E-08 | DOWN | GO:0050896 | autophagy-related protein |  |  |
| Ao3042_09533 | 139.6309 | 22.83784 | 2.6987 | 2.63E-32 | UP | GO:0050896 | RTA1 domain protein |  |  |
| Ao3042_09578 | 150.8028 | 42.40244 | 1.917 | 3.33E-53 | UP | GO:0050896 | sensory transduction histidine kinase |  |  |
| Ao3042_09584 | 118.5636 | 13.20986 | 3.2526 | 4.59E-34 | UP | GO:0050896 | cytochrome protein |  |  |
| Ao3042_09589 | 124.0747 | 26.11598 | 2.3348 | 5.35E-27 | UP | GO:0050896 | hypothetical protein |  |  |
| Ao3042_09710 | 16.22205 | 1.30206 | 3.7257 | 0.00032832 | UP | GO:0050896 | hypothetical protein |  |  |
| Ao3042_09842 | 523.8362 | 1128.695 | -1.0209 | 6.09E-48 | DOWN | GO:0050896 | hypothetical protein |  |  |
| Ao3042_10251 | 17.5154 | 7.238302 | 1.3615 | 2.42E-05 | UP | GO:0050896 | oxoprolinase |  |  |
| Ao3042_10327 | 24.59001 | 131.6143 | -2.3336 | 1.08E-24 | DOWN | GO:0050896 | catalase | |  |
| Ao3042_10361 | 64.61863 | 33.03297 | 1.0546 | 0.00040748 | UP | GO:0050896 | Zn-dependent hydrolase |  |  |
| Ao3042_10473 | 1.801212 | 8.217224 | -2.1031 | 8.93E-06 | DOWN | GO:0050896 | multidrug/pheromone exporter%2C ABC superfamily |  |  |
| Ao3042_10511 | 0.413018 | 3.768349 | -3.1031 | 0.00097155 | DOWN | GO:0050896 | RNA-directed RNA polymerase QDE-1 |  |  |
| Ao3042_10571 | 9.459608 | 20.25818 | -1.0121 | 8.12E-07 | DOWN | GO:0050896 | AAA+-type ATPase containing the bromodomain protein |  |  |
| Ao3042_10794 | 108.3176 | 27.53055 | 2.0627 | 2.16E-06 | UP | GO:0050896 | hypothetical protein |  |  |
| Ao3042_11018 | 7.48949 | 17.992 | -1.1778 | 0.00013584 | DOWN | GO:0050896 | translesion DNA polymerase - REV1 deoxycytidyl transferase |  |  |
| Ao3042_11081 | 47.14904 | 114.8605 | -1.198 | 1.29E-33 | DOWN | GO:0050896 | ribosomal protein S6 kinase |  |  |
| Ao3042_11245 | 38.24633 | 340.1429 | -3.0662 | 5.54E-74 | DOWN | GO:0050896 | conserved histidine-rich protein |  |  |
| Ao3042_11370 | 54.35763 | 7.317501 | 2.9796 | 2.56E-13 | UP | GO:0050896 | dehydrogenase with different specificitie |  |  |
| Ao3042_11627 | 15.26784 | 39.19645 | -1.2736 | 4.60E-11 | DOWN | GO:0050896 | acyl-CoA diacylglycerol acyltransferase |  |  |
| Ao3042_11750 | 7.774563 | 145.0579 | -4.1351 | 4.90E-36 | DOWN | GO:0050896 | hypothetical protein |  |  |
| Ao3042_11909 | 23.38467 | 58.08965 | -1.2261 | 0.00024325 | DOWN | GO:0050896 | hypothetical protein |  |  |
| Novel00051 | 60.28222 | 12.31977 | 2.3773 | 1.77E-05 | UP | GO:0050896 | hypothetical protein AOR_1_196074 |  |  |
| Novel00180 | 9.484899 | 59.52447 | -2.5632 | 3.52E-25 | DOWN | GO:0050896 | hypothetical protein AOR_1_2744154 |  |  |
| Novel00204 | 2.169789 | 13.09592 | -2.5069 | 0.00045375 | DOWN | GO:0050896 | phosphatidylserine decarboxylase family protein |  |  |
| Novel00770 | 211.6775 | 73.07099 | 1.6211 | 1.50E-17 | UP | GO:0050896 | THUMP domain protein |  |  |
| Novel00853 | 2.197197 | 377.8801 | -7.3395 | 0 | DOWN | GO:0050896 | hypothetical protein P034_00794513 |  |  |
| Novel00949 | 15.3781 | 1.81118 | 3.1725 | 1.01E-05 | UP | GO:0050896 | C-14 sterol reductase [Aspergillus oryzae RIB40] |  |  |
| Novel01036 | 1.087045 | 856.9573 | -9.5361 | 0 | DOWN | GO:0050896 | hypothetical protein PMG11_04269 |  |  |
| Novel01042 | 11.38772 | 37.03503 | -1.6148 | 0.00013076 | DOWN | GO:0050896 | hypothetical protein AKAW_07667 |  |  |
| Novel01257 | 18.0046 | 50.5227 | -1.402 | 1.99E-18 | DOWN | GO:0050896 | hypothetical protein AOR_1_1218164 |  |  |
