# Supplementary material for: Transcriptomic analysis reveals Aspergillus oryzae responds to temperature stress by regulating sugar metabolism and lipid metabolism
Source: PLoS One. 2022 Sep 12;17(9):e0274394. doi: 10.1371/journal.pone.0274394 (PMC9467314; doi:10.1371/journal.pone.0274394)
Supplement: S4 Table — (DOCX) [file pone.0274394.s008.docx]

| **S4 Table The details of DEGs in the GO term of response to stimulus in HT-vs-CK** | | | | | | | |  |  |  |  |
| --- | --- | --- | --- | --- | --- | --- | --- | --- | --- | --- | --- |
| Gene_id | HT_fpkm | CK_fpkm | log_2_FoldChange | pval | significant | biological_process | gene_description |  |  |  |  |
| Ao3042_07339 | 47.21034 | 0.731662 | 5.9417 | 1.80E-45 | UP | GO:0050896 | multidrug/pheromone exporter ABC superfamily |  |  |  |  |
| Ao3042_06529 | 266.496 | 4.167586 | 5.9287 | 3.09E-48 | UP | GO:0050896 | hypothetical protein |  |  |  |  |
| Ao3042_04094 | 18.32701 | 1.862883 | 3.2283 | 8.14E-06 | UP | GO:0050896 | spherulin 4-like cell surface protein |  |  |  |  |
| Ao3042_05436 | 91.93544 | 20.60947 | 2.0872 | 6.00E-28 | UP | GO:0050896 | hypothetical protein |  |  |  |  |
| Ao3042_07717 | 36.07878 | 8.244549 | 2.0595 | 2.53E-11 | UP | GO:0050896 | a-pheromone receptor PreA |  |  |  |  |
| Ao3042_06225 | 179.3819 | 54.53213 | 1.6478 | 1.44E-31 | UP | GO:0050896 | xanthine/uracil permease |  |  |  |  |
| Ao3042_07099 | 289.2092 | 90.37594 | 1.608 | 4.24E-32 | UP | GO:0050896 | RTA1 domain protein |  |  |  |  |
| Ao3042_05449 | 327.9761 | 104.7026 | 1.5772 | 4.72E-84 | UP | GO:0050896 | allergen Asp f 7 |  |  |  |  |
| Ao3042_09046 | 32.35737 | 10.50086 | 1.5535 | 7.41E-06 | UP | GO:0050896 | hypothetical protein |  |  |  |  |
| Ao3042_05469 | 24.06781 | 8.042987 | 1.5112 | 4.57E-10 | UP | GO:0050896 | dDENN domain protein |  |  |  |  |
| Ao3042_07422 | 85.62051 | 28.75757 | 1.5039 | 2.22E-05 | UP | GO:0050896 | hypothetical protein |  |  |  |  |
| Ao3042_06402 | 9.83169 | 3.334517 | 1.4899 | 0.000135 | UP | GO:0050896 | hypothetical protein |  |  |  |  |
| Ao3042_08724 | 49.31802 | 17.21884 | 1.448 | 5.36E-09 | UP | GO:0050896 | dihydroxy-acid dehydratase |  |  |  |  |
| Ao3042_05564 | 58.79414 | 22.69173 | 1.3034 | 3.71E-14 | UP | GO:0050896 | hypothetical protein |  |  |  |  |
| Novel00392 | 216.9553 | 92.13138 | 1.1655 | 7.00E-61 | UP | GO:0050896 | S1S28E like protein |  |  |  |  |
| Ao3042_03236 | 32.16118 | 13.85384 | 1.1449 | 5.42E-05 | UP | GO:0050896 | hypothetical protein |  |  |  |  |
| Ao3042_00048 | 38.25999 | 16.71454 | 1.1246 | 1.08E-05 | UP | GO:0050896 | alpha-glucosidase |  |  |  |  |
| Ao3042_00791 | 15.95141 | 6.990095 | 1.1202 | 0.00099 | UP | GO:0050896 | asparaginase |  |  |  |  |
| Ao3042_01246 | 69.48083 | 30.81126 | 1.1031 | 6.08E-08 | UP | GO:0050896 | amino acid transporter |  |  |  |  |
| Ao3042_09508 | 26.57626 | 11.93223 | 1.0852 | 6.28E-06 | UP | GO:0050896 | vacuolar sorting protein VPS1 dynamin |  |  |  |  |
| Ao3042_05274 | 23.12168 | 10.51368 | 1.0669 | 0.001318 | UP | GO:0050896 | Tyrosyl-tRNA synthetase |  |  |  |  |
| Ao3042_02600 | 82.12931 | 156.8965 | -1.0039 | 2.16E-29 | DOWN | GO:0050896 | phosphotransmitter protein Ypd1 putative |  |  |  |  |
| Ao3042_09072 | 24.67505 | 47.1713 | -1.0049 | 0.000171 | DOWN | GO:0050896 | UV damage repair endonuclease |  |  |  |  |
| Ao3042_06045 | 70.11996 | 137.224 | -1.0387 | 9.61E-16 | DOWN | GO:0050896 | hypothetical protein |  |  |  |  |
| Ao3042_05378 | 18.43879 | 36.14123 | -1.041 | 4.36E-06 | DOWN | GO:0050896 | ubiquitin fusion degradation protein-2 |  |  |  |  |
| Ao3042_07180 | 116.2845 | 228.085 | -1.042 | 8.04E-31 | DOWN | GO:0050896 | hypothetical protein |  |  |  |  |
| Ao3042_11750 | 73.16849 | 145.0579 | -1.0574 | 2.84E-07 | DOWN | GO:0050896 | hypothetical protein |  |  |  |  |
| Ao3042_06147 | 15.21189 | 30.42883 | -1.0703 | 3.39E-07 | DOWN | GO:0050896 | bacteriophytochrome |  |  |  |  |
| Ao3042_04764 | 32.27802 | 64.8784 | -1.0773 | 7.55E-08 | DOWN | GO:0050896 | peroxisomal long-chain acyl-CoA transporter ABC superfamily |  |  |  |  |
| Ao3042_01012 | 22.66521 | 45.64041 | -1.0799 | 0.000637 | DOWN | GO:0050896 | erythromycin esterase like protein |  |  |  |  |
| Ao3042_06581 | 23.74435 | 47.95059 | -1.0841 | 7.76E-05 | DOWN | GO:0050896 | GTPase Rab5/YPT51 |  |  |  |  |
| Ao3042_11245 | 167.9873 | 340.1429 | -1.0879 | 1.83E-17 | DOWN | GO:0050896 | conserved histidine-rich protein |  |  |  |  |
| Ao3042_03262 | 28.21211 | 57.59425 | -1.0997 | 5.98E-08 | DOWN | GO:0050896 | MAPKKK cascade protein kinase regulator Ste50 |  |  |  |  |
| Ao3042_00419 | 12.05166 | 25.0655 | -1.1266 | 0.000111 | DOWN | GO:0050896 | ATPases of the AAA+ class |  |  |  |  |
| Ao3042_07238 | 85.49812 | 179.0191 | -1.1362 | 5.08E-12 | DOWN | GO:0050896 | ubiquitin-like protein |  |  |  |  |
| Ao3042_02440 | 33.07059 | 69.80089 | -1.1478 | 4.20E-13 | DOWN | GO:0050896 | arginine/serine-rich splicing factor putative |  |  |  |  |
| Ao3042_08515 | 6.736827 | 14.3348 | -1.1595 | 0.000127 | DOWN | GO:0050896 | pleiotropic drug resistance proteins (PDR1-15) ABC superfamily |  |  |  |  |
| Ao3042_02046 | 27.76232 | 59.84368 | -1.1782 | 9.35E-08 | DOWN | GO:0050896 | phospholipase A2-activating protein |  |  |  |  |
| Ao3042_07695 | 19.83321 | 44.68299 | -1.2419 | 5.30E-05 | DOWN | GO:0050896 | cell division cycle 37 protein CDC37 |  |  |  |  |
| Ao3042_01093 | 14.52924 | 33.03924 | -1.2553 | 0.000363 | DOWN | GO:0050896 | protein involved in cell differentiation/sexual development |  |  |  |  |
| Ao3042_02036 | 20.52116 | 47.60407 | -1.2841 | 3.39E-09 | DOWN | GO:0050896 | hypothetical protein |  |  |  |  |
| Ao3042_09234 | 13.78304 | 32.0359 | -1.2869 | 0.000446 | DOWN | GO:0050896 | autophagy-related protein |  |  |  |  |
| Ao3042_05005 | 86.35947 | 201.2463 | -1.2906 | 1.63E-06 | DOWN | GO:0050896 | hypothetical protein |  |  |  |  |
| Ao3042_00276 | 1720.316 | 4026.856 | -1.2971 | 2.55E-165 | DOWN | GO:0050896 | hypothetical protein |  |  |  |  |
| Ao3042_05395 | 29.01218 | 68.47893 | -1.3091 | 3.60E-14 | DOWN | GO:0050896 | nuclear envelope protein |  |  |  |  |
| Ao3042_10571 | 8.458877 | 20.25818 | -1.3301 | 3.79E-08 | DOWN | GO:0050896 | AAA+-type ATPase containing the bromodomain protein |  |  |  |  |
| Ao3042_07077 | 7.86143 | 19.24362 | -1.3616 | 1.30E-05 | DOWN | GO:0050896 | mRNA cleavage and polyadenylation factor I complex subunit RNA14 |  |  |  |  |
| Ao3042_07150 | 18.55805 | 46.44715 | -1.3936 | 1.25E-08 | DOWN | GO:0050896 | galactokinase |  |  |  |  |
| Ao3042_03264 | 124.4244 | 316.9489 | -1.4191 | 4.89E-88 | DOWN | GO:0050896 | AAA+-type ATPase |  |  |  |  |
| Ao3042_00432 | 10.03031 | 25.7091 | -1.428 | 5.63E-10 | DOWN | GO:0050896 | hypothetical protein |  |  |  |  |
| Ao3042_11143 | 18.07598 | 47.22841 | -1.4557 | 1.11E-05 | DOWN | GO:0050896 | protocatechuate 3 4-dioxygenase beta subunit |  |  |  |  |
| Novel00853 | 144.5143 | 377.8801 | -1.4568 | 2E-113 | DOWN | GO:0050896 | hypothetical protein P034_00794513 |  |  |  |  |
| Ao3042_07031 | 7.434499 | 20.20125 | -1.5122 | 5.59E-06 | DOWN | GO:0050896 | hypothetical protein |  |  |  |  |
| Novel01036 | 314.4324 | 856.9573 | -1.5166 | 3.66E-135 | DOWN | GO:0050896 | hypothetical protein PMG11_04269 |  |  |  |  |
| Ao3042_01173 | 21.78677 | 59.61964 | -1.5224 | 2.53E-05 | DOWN | GO:0050896 | hypothetical protein |  |  |  |  |
| Ao3042_04131 | 397.4424 | 1091.109 | -1.5271 | 9.95E-53 | DOWN | GO:0050896 | hypothetical protein |  |  |  |  |
| Ao3042_04375 | 9.347885 | 25.92811 | -1.5419 | 2.02E-07 | DOWN | GO:0050896 | aspartic endopeptidase |  |  |  |  |
| Ao3042_07654 | 15.33181 | 43.42998 | -1.5723 | 9.10E-12 | DOWN | GO:0050896 | signal transduction histidine kinase |  |  |  |  |
| Ao3042_09922 | 26.19847 | 74.59926 | -1.5798 | 1.19E-38 | DOWN | GO:0050896 | sensory transduction histidine kinase |  |  |  |  |
| Ao3042_01245 | 289.6109 | 837.5431 | -1.6021 | 2.26E-131 | DOWN | GO:0050896 | alcohol dehydrogenase class V |  |  |  |  |
| Ao3042_06464 | 43.65254 | 128.8907 | -1.6321 | 1.30E-38 | DOWN | GO:0050896 | monodehydroascorbate/ferredoxin reductase |  |  |  |  |
| Ao3042_05775 | 54.71327 | 170.0637 | -1.7062 | 3.04E-13 | DOWN | GO:0050896 | hypothetical protein |  |  |  |  |
| Novel01042 | 11.87969 | 37.03503 | -1.7105 | 0.000194 | DOWN | GO:0050896 | hypothetical protein AKAW_07667 |  |  |  |  |
| Ao3042_09900 | 154.6158 | 486.6635 | -1.7243 | 2.11E-24 | DOWN | GO:0050896 | glutathione peroxidase |  |  |  |  |
| Ao3042_06040 | 38.78389 | 123.7041 | -1.7435 | 4.27E-11 | DOWN | GO:0050896 | putative Zn-finger protein |  |  |  |  |
| Ao3042_07653 | 30.98628 | 105.745 | -1.841 | 2.73E-05 | DOWN | GO:0050896 | hypothetical protein |  |  |  |  |
| Ao3042_04363 | 9.88091 | 34.56504 | -1.8767 | 5.44E-21 | DOWN | GO:0050896 | hypothetical protein |  |  |  |  |
| Ao3042_04727 | 5.816232 | 20.68663 | -1.9006 | 2.23E-11 | DOWN | GO:0050896 | phosphoinositide-specific phospholipase C |  |  |  |  |
| Ao3042_07095 | 121.9282 | 433.8627 | -1.9013 | 3.29E-32 | DOWN | GO:0050896 | glutathione S-transferase |  |  |  |  |
| Ao3042_02691 | 52.22305 | 186.7321 | -1.9083 | 1.62E-29 | DOWN | GO:0050896 | Aha1 domain family |  |  |  |  |
| Ao3042_06583 | 22.49773 | 82.92017 | -1.952 | 3.79E-46 | DOWN | GO:0050896 | hypothetical protein |  |  |  |  |
| Ao3042_11401 | 288.3069 | 1141.823 | -2.0558 | ###### | DOWN | GO:0050896 | molecular chaperone |  |  |  |  |
| Ao3042_09113 | 47.35329 | 209.9176 | -2.2184 | 1.39E-43 | DOWN | GO:0050896 | glutathione S-transferase |  |  |  |  |
| Ao3042_03618 | 19.1704 | 85.03397 | -2.2192 | 2.02E-16 | DOWN | GO:0050896 | hypothetical protein |  |  |  |  |
| Ao3042_09584 | 2.568822 | 13.20986 | -2.4325 | 0.000495 | DOWN | GO:0050896 | cytochrome protein |  |  |  |  |
| Ao3042_00675 | 8.085497 | 42.63216 | -2.4686 | 3.34E-11 | DOWN | GO:0050896 | AAA+-type ATPase |  |  |  |  |
| Ao3042_01627 | 1.149384 | 6.437385 | -2.5557 | 0.000201 | DOWN | GO:0050896 | LEA domain protein |  |  |  |  |
| Ao3042_09589 | 4.564893 | 26.11598 | -2.5864 | 1.34E-07 | DOWN | GO:0050896 | hypothetical protein |  |  |  |  |
| Ao3042_07384 | 1.810647 | 16.08891 | -3.2216 | 0.000431 | DOWN | GO:0050896 | hypothetical protein |  |  |  |  |
| Ao3042_09106 | 2.820706 | 27.35824 | -3.3479 | 0.000777 | DOWN | GO:0050896 | hypothetical protein |  |  |  |  |
| Ao3042_08157 | 3.08768 | 42.94886 | -3.8681 | 9.16E-22 | DOWN | GO:0050896 | catalase | | |  |  |
| Ao3042_10355 | 18.61758 | 396.1135 | -4.4813 | 8.29E-163 | DOWN | GO:0050896 | hypothetical protein |  |  |  |  |
| Ao3042_08612 | 14.28645 | 750.2865 | -5.7848 | 0 | DOWN | GO:0050896 | vacuolar sorting protein VPS1 dynamin |  |  |  |  |
| Novel01343 | 21.98267 | 1209.377 | -5.8518 | 6.1E-171 | DOWN | GO:0050896 | hypothetical protein AO1008_10115 |  |  |  |  |
