# Supplementary material for: Transcriptomic analysis reveals Aspergillus oryzae responds to temperature stress by regulating sugar metabolism and lipid metabolism
Source: PLoS One. 2022 Sep 12;17(9):e0274394. doi: 10.1371/journal.pone.0274394 (PMC9467314; doi:10.1371/journal.pone.0274394)
Supplement: S5 Table — (DOCX) [file pone.0274394.s009.docx]

**S5 Table The expression levels of DEGs in sugar mechanism**

| Gene_id | LT_fpkm | HT_fpkm | CK_fpkm | log_2_FoldChange  (HTvsCK) | Significant  (HTvsCK) | log_2_FoldChange  (LTvsCK) | Significant  (LTvsCK) | KEGG Annotated Information |
| --- | --- | --- | --- | --- | --- | --- | --- | --- |
| **Starch and sucrose metabolism** | | | | | | | | |
| Ao3042_01611 | 3.34787 | 26.312844 | 20.61754 | 0.2818 | - | -2.536 | DOWN | K00012 UDPglucose 6-dehydrogenase [EC:1.1.1.22] |
| Ao3042_04763 | 270.824 | 11.302458 | 107.7329 | -3.3228 | DOWN | 1.4165 | UP | K00688 glycogen phosphorylase [EC:2.4.1.1] |
| Ao3042_06668 | 31.7183 | 17.730941 | 48.32291 | -1.5165 | DOWN | -0.52081 | - | K00693 glycogen synthase [EC:2.4.1.11] |
| Ao3042_07284 | 93.0438 | 86.584782 | 210.1918 | -1.3496 | DOWN | -1.0891 | DOWN | K00697 trehalose 6-phosphate synthase [EC:2.4.1.15] |
| Ao3042_03762 | 113.306 | 68.218302 | 188.3936 | -1.5356 | DOWN | -0.64695 | - | K00697 trehalose 6-phosphate synthase [EC:2.4.1.15] |
| Ao3042_03310 | 2.54445 | 46.257628 | 91.02831 | -1.0467 | DOWN | -5.0743 | DOWN | K00697 trehalose 6-phosphate synthase [EC:2.4.1.15] |
| Ao3042_04331 | 186.872 | 150.34748 | 93.57682 | 0.61399 | - | 1.0844 | UP | K00706 1,3-beta-glucan synthase [EC:2.4.1.34] |
| Ao3042_10845 | 404.302 | 202.74384 | 398.8771 | -1.0464 | DOWN | 0.10607 | - | K00844 hexokinase [EC:2.7.1.1] |
| Ao3042_03901 | 6.86054 | 34.842883 | 14.863 | 1.159 | UP | -1.0287 | - | K00844 hexokinase [EC:2.7.1.1] |
| Ao3042_03902 | 5.30813 | 30.57708 | 11.49165 | 1.3418 | UP | -1.0277 | - | K00844 hexokinase [EC:2.7.1.1] |
| Ao3042_00337 | 236.66 | 103.78968 | 240.9444 | -1.2851 | DOWN | 0.060698 | - | K00844 hexokinase [EC:2.7.1.1] |
| Ao3042_10648 | 12.5274 | 22.625039 | 27.76879 | -0.36563 | - | -1.0618 | DOWN | K01178 glucoamylase [EC:3.2.1.3] |
| Ao3042_08133 | 22.6828 | 25.407829 | 6.029447 | 2.0051 | UP | 1.9981 | UP | K01178 glucoamylase [EC:3.2.1.3] |
| Ao3042_05624 | 705.567 | 10.330345 | 85.95225 | -3.1267 | DOWN | 3.1238 | UP | K01179 endoglucanase [EC:3.2.1.4] |
| Ao3042_07614 | 11.4202 | 57.330241 | 39.0983 | 0.4821 | - | -1.6889 | DOWN | K01182 oligo-1,6-glucosidase [EC:3.2.1.10] |
| Ao3042_08758 | 29.9304 | 162.66854 | 24.37863 | 2.6682 | UP | 0.38258 | - | K01184 polygalacturonase [EC:3.2.1.15] |
| Ao3042_11801 | 50.9297 | 10.070579 | 14.55563 | -0.60152 | - | 1.8935 | UP | K01187 alpha-glucosidase [EC:3.2.1.20] |
| Ao3042_07367 | 29.73 | 3.98814 | 3.858012 | -0.022232 | - | 3.0326 | UP | K01193 beta-fructofuranosidase [EC:3.2.1.26] |
| Ao3042_04366 | 76.2633 | 82.427653 | 161.3327 | -1.0389 | DOWN | -0.99439 | - | K01194 alpha,alpha-trehalase [EC:3.2.1.28] |
| Ao3042_03419 | 58.9318 | 33.374173 | 84.55651 | -1.4113 | DOWN | -0.43429 | - | K01196 glycogen debranching enzyme [EC:2.4.1.25 3.2.1.33] |
| Ao3042_00156 | 21.5392 | 25.695942 | 66.80003 | -1.4484 | DOWN | -1.5463 | DOWN | K01210 glucan 1,3-beta-glucosidase [EC:3.2.1.58] |
| Ao3042_08961 | 12.3782 | 12.615075 | 1.949912 | 2.6236 | UP | 2.7529 | UP | K01213 galacturan 1,4-alpha-galacturonidase [EC:3.2.1.67] |
| Ao3042_01015 | 23.6094 | 20.997168 | 53.42527 | -1.4174 | DOWN | -1.0916 | DOWN | K05349 beta-glucosidase [EC:3.2.1.21] |
| Ao3042_02850 | 19.3966 | 10.075304 | 5.03536 | 0.93057 | UP | 2.0322 | UP | K05349 beta-glucosidase [EC:3.2.1.21] |
| Ao3042_03761 | 61.2671 | 36.339276 | 98.02777 | -1.5018 | DOWN | -0.59149 | - | K16055 trehalose 6-phosphate synthase/phosphatase [EC:2.4.1.15 3.1.3.12] |
| Ao3042_11651 | 66.5492 | 54.14481 | 129.8075 | -1.3316 | DOWN | -0.8773 | - | K16055 trehalose 6-phosphate synthase/phosphatase [EC:2.4.1.15 3.1.3.12] |
| **Fructose and mannose metabolism** | | | | | | | | |
| Ao3042_11806 | 78.8732 | 39.168795 | 99.0131 | -1.408 | DOWN | -0.2415 | - | K00008 L-iditol 2-dehydrogenase [EC:1.1.1.14] |
| Ao3042_09851 | 575.373 | 578.03401 | 1344.703 | -1.2882 | DOWN | -1.1381 | DOWN | K00009 mannitol-1-phosphate 5-dehydrogenase [EC:1.1.1.17] |
| Ao3042_10845 | 404.302 | 202.74384 | 398.8771 | -1.0464 | DOWN | 0.10607 | - | K00844 hexokinase [EC:2.7.1.1] |
| Ao3042_03901 | 6.86054 | 34.842883 | 14.863 | 1.159 | UP | -1.0287 | - | K00844 hexokinase [EC:2.7.1.1] |
| Ao3042_03902 | 5.30813 | 30.57708 | 11.49165 | 1.3418 | UP | -1.0277 | - | K00844 hexokinase [EC:2.7.1.1] |
| Ao3042_00337 | 236.66 | 103.78968 | 240.9444 | -1.2851 | DOWN | 0.060698 | - | K00844 hexokinase [EC:2.7.1.1] |
| Ao3042_08394 | 241.243 | 31.498216 | 116.3976 | -1.9558 | DOWN | 1.138 | UP | K00850 6-phosphofructokinase 1 [EC:2.7.1.11] |
| Ao3042_03218 | 12.2002 | 14.4192 | 34.56779 | -1.3315 | DOWN | -1.4159 | DOWN | K00900 6-phosphofructo-2-kinase [EC:2.7.1.105] |
| Ao3042_04078 | 905.363 | 467.8284 | 1061.934 | -1.2527 | DOWN | -0.14354 | - | K01803 triosephosphate isomerase (TIM) [EC:5.3.1.1] |
| Ao3042_05664 | 428.052 | 185.44495 | 193.0787 | -0.12829 | - | 1.2352 | UP | K01808 ribose 5-phosphate isomerase B [EC:5.3.1.6] |
| Ao3042_04724 | 126.991 | 55.838569 | 108.8186 | -1.0327 | DOWN | 0.30938 | - | K01809 mannose-6-phosphate isomerase [EC:5.3.1.8] |
| Ao3042_05265 | 11.4727 | 78.024189 | 125.7564 | -0.75873 | - | -3.3678 | DOWN | K01809 mannose-6-phosphate isomerase [EC:5.3.1.8] |
| Ao3042_05291 | 46.334 | 5.2602967 | 8.524813 | -0.76661 | - | 2.5289 | UP | K17742 sorbose reductase [EC:1.1.1.289] |
| **Galactose metabolism** | | | | | | | | |
| Ao3042_10845 | 404.302 | 202.74384 | 398.8771 | -1.0464 | DOWN | 0.10607 | - | K00844 hexokinase [EC:2.7.1.1] |
| Ao3042_03902 | 5.30813 | 30.57708 | 11.49165 | 1.3418 | UP | -1.0277 | - | K00844 hexokinase [EC:2.7.1.1] |
| Ao3042_03901 | 6.86054 | 34.842883 | 14.863 | 1.159 | UP | -1.0287 | - | K00844 hexokinase [EC:2.7.1.1] |
| Ao3042_00337 | 236.66 | 103.78968 | 240.9444 | -1.2851 | DOWN | 0.060698 | - | K00844 hexokinase [EC:2.7.1.1] |
| Ao3042_07150 | 72.377 | 18.55805 | 46.44715 | -1.3936 | DOWN | 0.72653 | - | K00849 galactokinase [EC:2.7.1.6] |
| Ao3042_08394 | 241.243 | 31.498216 | 116.3976 | -1.9558 | DOWN | 1.138 | UP | K00850 6-phosphofructokinase 1 [EC:2.7.1.11] |
| Ao3042_05709 | 74.1651 | 43.827779 | 86.7598 | -1.0553 | DOWN | -0.1397 | - | K00965 UDPglucose--hexose-1-phosphate uridylyltransferase [EC:2.7.7.12] |
| Ao3042_07614 | 11.4202 | 57.330241 | 39.0983 | 0.4821 | - | -1.6889 | DOWN | K01182 oligo-1,6-glucosidase [EC:3.2.1.10] |
| Ao3042_11801 | 50.9297 | 10.070579 | 14.55563 | -0.60152 | - | 1.8935 | UP | K01187 alpha-glucosidase [EC:3.2.1.20] |
| Ao3042_07367 | 29.73 | 3.98814 | 3.858012 | -0.022232 | - | 3.0326 | UP | K01193 beta-fructofuranosidase [EC:3.2.1.26] |
| Ao3042_03798 | 100.054 | 50.132998 | 18.48275 | 1.3695 | UP | 2.5231 | UP | K01784 UDP-glucose 4-epimerase [EC:5.1.3.2] |
| Ao3042_08380 | 423.241 | 164.55671 | 129.7654 | 0.27259 | - | 1.7922 | UP | K01784 UDP-glucose 4-epimerase [EC:5.1.3.2] |
| Novel00952 | 1.8050651 | 0.1128282 | 0.174701 | -0.70085 | - | 3.4557 | Up | K07407 alpha-galactosidase [EC:3.2.1.22] |
| Ao3042_08190 | 1.8186421 | 0.1757459 | 0.18808 | -0.30392 | - | 1.4888 | Up | K07407 alpha-galactosidase [EC:3.2.1.22] |
