# Supplementary material for: Transcriptomic analysis reveals Aspergillus oryzae responds to temperature stress by regulating sugar metabolism and lipid metabolism
Source: PLoS One. 2022 Sep 12;17(9):e0274394. doi: 10.1371/journal.pone.0274394 (PMC9467314; doi:10.1371/journal.pone.0274394)
Supplement: S6 Table — (DOCX) [file pone.0274394.s010.docx]

**S6 Table The expression levels of DEGs in glycerophospholipid and glycerolipid metabolism**

| Gene_id | LT_fpkm | | HT_fpkm | | CK_fpkm | | log2FoldChange  (HTvsCK) | | Significant  (HTvsCK) | | log2FoldChange  (LTvsCK) | | Significant  (LTvsCK) | | KEGG Annotated Information | |  |
| --- | --- | --- | --- | --- | --- | --- | --- | --- | --- | --- | --- | --- | --- | --- | --- | --- | --- |
| Glycerophospholipid metabolism | | | | | |  | |  | |  | |  | |  | |  | |
| Ao3042_00793 | | 9.606618 | | 65.47671 | | 46.90822 | | 0.41105 | | - | | -2.2012 | | DOWN | | K18696 glycerophosphodiester phosphodiesterase [EC:3.1.4.46] | |
| Ao3042_01402 | | 0.440512 | | 37.772 | | 3.005059 | | 3.5818 | | UP | | -2.6836 | | - | | K00134 glyceraldehyde 3-phosphate dehydrogenase [EC:1.2.1.12] | |
| Ao3042_02083 | | 6.056647 | | 183.1124 | | 503.584 | | -1.5296 | | DOWN | | -6.291 | | DOWN | | K01613 phosphatidylserine decarboxylase [EC:4.1.1.65] | |
| Ao3042_02694 | | 35.73288 | | 32.28231 | | 83.79414 | | -1.4462 | | DOWN | | -1.143 | | DOWN | | K13507 glycerol-3-phosphate O-acyltransferase / dihydroxyacetone phosphate acyltransferase [EC:2.3.1.15] | |
| Ao3042_03065 | | 116.1521 | | 56.61396 | | 55.47764 | | -0.040838 | | - | | 1.1526 | | UP | | K13333 lysophospholipase [EC:3.1.1.5] | |
| Ao3042_04827 | | 27.22861 | | 35.6273 | | 4.733542 | | 2.8419 | | UP | | 2.6107 | | UP | | K13333 lysophospholipase [EC:3.1.1.5] | |
| Ao3042_07857 | | 17.34378 | | 12.00587 | | 24.4262 | | -1.0948 | | DOWN | | -0.40743 | | - | | K14676 lysophospholipid hydrolase [EC:3.1.1.5] | |
| Ao3042_04357 | | 169.0064 | | 490.6173 | | 389.9944 | | 0.26105 | | - | | -1.1198 | | DOWN | | K17103 CDP-diacylglycerol---serine O-phosphatidyltransferase [EC:2.7.8.8] | |
| Ao3042_04447 | | 142.4362 | | 64.27185 | | 146.515 | | -1.2589 | | DOWN | | 0.045851 | | - | | K00111 glycerol-3-phosphate dehydrogenase [EC:1.1.5.3] | |
| Ao3042_04838 | | 37.61866 | | 33.36957 | | 13.44911 | | 1.2409 | | UP | | 1.5705 | | UP | | K06123 1-acylglycerone phosphate reductase [EC:1.1.1.101] | |
| Ao3042_05032 | | 19.38733 | | 28.07139 | | 88.86865 | | -1.7327 | | DOWN | | -2.11 | | DOWN | | K00006 glycerol-3-phosphate dehydrogenase (NAD+) [EC:1.1.1.8] | |
| Ao3042_05652 | | 80.32609 | | 258.8774 | | 115.6641 | | 1.0922 | | UP | | -0.43942 | | - | | K13621 betaine lipid synthase | |
| Ao3042_05653 | | 19.25259 | | 52.03767 | | 14.71538 | | 1.7521 | | UP | | 0.47431 | | - | | K13621 betaine lipid synthase | |
| Ao3042_05741 | | 32.41316 | | 74.49978 | | 105.4341 | | -0.57112 | | - | | -1.6151 | | DOWN | | K16342 cytosolic phospholipase A2 [EC:3.1.1.4] | |
| Ao3042_06209 | | 5.860505 | | 38.12644 | | 5.54328 | | 2.7119 | | UP | | 0.16687 | | - | | K01126 glycerophosphoryl diester phosphodiesterase [EC:3.1.4.46] | |
| Ao3042_06500 | | 6.698519 | | 31.13718 | | 46.10302 | | -0.63631 | | - | | -2.6964 | | DOWN | | K01613 phosphatidylserine decarboxylase [EC:4.1.1.65] | |
| Ao3042_07786 | | 49.98156 | | 153.3251 | | 27.68512 | | 2.3993 | | UP | | 0.93887 | | - | | K01114 phospholipase C [EC:3.1.4.3] | |
| Ao3042_10549 | | 56.26167 | | 250.9006 | | 48.75534 | | 2.2934 | | UP | | 0.29318 | | - | | K01114 phospholipase C [EC:3.1.4.3] | |
| Ao3042_09717 | | 5398.419 | | 2516.852 | | 2599.235 | | -0.11656 | | - | | 1.141 | | UP | | K00134 glyceraldehyde 3-phosphate dehydrogenase [EC:1.2.1.12] | |
| Novel00204 | | 2.169789 | | 15.06103 | | 13.09592 | | 0.13161 | | - | | -2.5069 | | DOWN | | K01613 phosphatidylserine decarboxylase [EC:4.1.1.65] | |
| Ao3042_09236 | | 11.14416 | | 8.23047 | | 27.08149 | | -1.7884 | | DOWN | | -1.1944 | | DOWN | | K14674 1-acylglycerol-3phosphate acyltransferase [EC:2.3.1.51]/ triacylglycerol lipase [EC:[3.1.1.3](https://www.kegg.jp/entry/3.1.1.3)] | |
| Glycerolipid metabolism | | | |  | |  | |  | |  | |  | |  | |  | |
| Ao3042_00616 | | 64.63334 | | 25.02273 | | 67.54937 | | -1.5028 | | DOWN | | 0.02292 | | - | | K00863 triose/dihydroxyacetone kinase / FAD-AMP lyase (cyclizing) [2.7.1.29] | |
| Ao3042_02694 | | 35.73288 | | 32.28231 | | 83.79414 | | -1.4462 | | DOWN | | -1.143 | | DOWN | | K13507 glycerol-3-phosphate O-acyltransferase / dihydroxyacetone phosphate acyltransferase [EC:2.3.1.15] | |
| Ao3042_02707 | | 142.1796 | | 38.77509 | | 171.116 | | -2.2119 | | DOWN | | -0.18068 | | - | | K00864 glycerol kinase [EC:2.7.1.30] | |
| Ao3042_02917 | | 81.23478 | | 33.8892 | | 70.37077 | | -1.1242 | | DOWN | | 0.29371 | | - | | K00128 aldehyde dehydrogenase (NAD+) [EC:1.2.1.3] | |
| Ao3042_05018 | | 78.43831 | | 63.30287 | | 468.5994 | | -2.9581 | | DOWN | | -2.4921 | | DOWN | | K11155 diacylglycerol O-acyltransferase 1 [EC:2.3.1.20] | |
| Ao3042_10146 | | 6.160379 | | 90.85958 | | 34.62881 | | 1.3216 | | UP | | -2.4043 | | DOWN | | K07407 alpha-galactosidase [EC:3.2.1.22] | |
| Ao3042_09236 | | 11.14416 | | 8.23047 | | 27.08149 | | -1.7884 | | DOWN | | -1.1944 | | DOWN | | K14674 1-acylglycerol-3-phosphate acyltransferase [EC:2.3.1.51]/ triacylglycerol lipase [EC:[3.1.1.3](https://www.kegg.jp/entry/3.1.1.3)] | |
