# Supplementary material for: Transcriptomic analysis reveals Aspergillus oryzae responds to temperature stress by regulating sugar metabolism and lipid metabolism
Source: PLoS One. 2022 Sep 12;17(9):e0274394. doi: 10.1371/journal.pone.0274394 (PMC9467314; doi:10.1371/journal.pone.0274394)
Supplement: S7 Table — (DOCX) [file pone.0274394.s011.docx]

**S7 Table The expression levels of DEGs in linoleic acid metabolism**

| Gene_id | LT_fpkm | HT_fpkm | CK_fpkm | log2FoldChange  (HTvsCK) | Significant  (HTvsCK) | log2FoldChange  (LTvsCK) | Significant  (LTvsCK) | KEGG Annotated Information |
| --- | --- | --- | --- | --- | --- | --- | --- | --- |
| Ao3042_09236 | 11.1441591 | 8.23046965 | 27.0814918 | -1.7884 | Down | -1.1944 | Down | K14674 TAG lipase / steryl ester hydrolase / phospholipase A2 / LPA acyltransferase [EC: 3.1.1.4] |
| Ao3042_08269 | 0.03871328 | 11.2921384 | 47.3752449 | -2.1389 | Down | -10.171 | Down | K17862 linoleate 10R-lipoxygenase [EC:1.13.11.62] |
| Ao3042_05741 | 32.4131628 | 74.4997806 | 105.43413 | -0.57112 | - | -1.6151 | Down | K16342 cytosolic phospholipase A2 [EC:3.1.1.4] |
| Ao3042_02662 | 91.6758202 | 266.159388 | 423.323488 | -0.73956 | - | -2.1206 | Down | K07513 acetyl-CoA acyltransferase 1 [EC:2.3.1.16] |
